# Linear mtDNA fragments and unusual mtDNA rearrangements associated with pathological deficiency of MGME1 exonuclease

**DOI:** 10.1093/hmg/ddu336

**Published:** 2014-06-30

**Authors:** Thomas J. Nicholls, Gábor Zsurka, Viktoriya Peeva, Susanne Schöler, Roman J. Szczesny, Dominik Cysewski, Aurelio Reyes, Cornelia Kornblum, Monica Sciacco, Maurizio Moggio, Andrzej Dziembowski, Wolfram S. Kunz, Michal Minczuk

**Affiliations:** 1 Mitochondrial Biology Unit, Medical Research Council, Cambridge, UK,; 2 Department of Epileptology,; 3 Life and Brain Center and; 4 Department of Neurology, University of Bonn Medical Center, Bonn, Germany,; 5 Institute of Genetics and Biotechnology, Faculty of Biology, University of Warsaw, Warsaw, Poland,; 6 Institute of Biochemistry and Biophysics, Polish Academy of Sciences, Warsaw, Poland and; 7 Neuromuscular Unit, Fondazione IRCCS Ca’ Granda, Ospedale Maggiore Policlinico, Centro Dino Ferrari, University of Milan, Milan, Italy

## Abstract

MGME1, also known as Ddk1 or C20orf72, is a mitochondrial exonuclease found to be involved in the processing of mitochondrial DNA (mtDNA) during replication. Here, we present detailed insights on the role of MGME1 in mtDNA maintenance. Upon loss of MGME1, elongated 7S DNA species accumulate owing to incomplete processing of 5′ ends. Moreover, an 11-kb linear mtDNA fragment spanning the entire major arc of the mitochondrial genome is generated. In contrast to control cells, where linear mtDNA molecules are detectable only after nuclease S1 treatment, the 11-kb fragment persists in MGME1-deficient cells. In parallel, we observed characteristic mtDNA duplications in the absence of MGME1. The fact that the breakpoints of these mtDNA rearrangements do not correspond to either classical deletions or the ends of the linear 11-kb fragment points to a role of MGME1 in processing mtDNA ends, possibly enabling their repair by homologous recombination. In agreement with its functional involvement in mtDNA maintenance, we show that MGME1 interacts with the mitochondrial replicase PolgA, suggesting that it is a constituent of the mitochondrial replisome, to which it provides an additional exonuclease activity. Thus, our results support the viewpoint that MGME1-mediated mtDNA processing is essential for faithful mitochondrial genome replication and might be required for intramolecular recombination of mtDNA.

## INTRODUCTION

**Figure 1.**
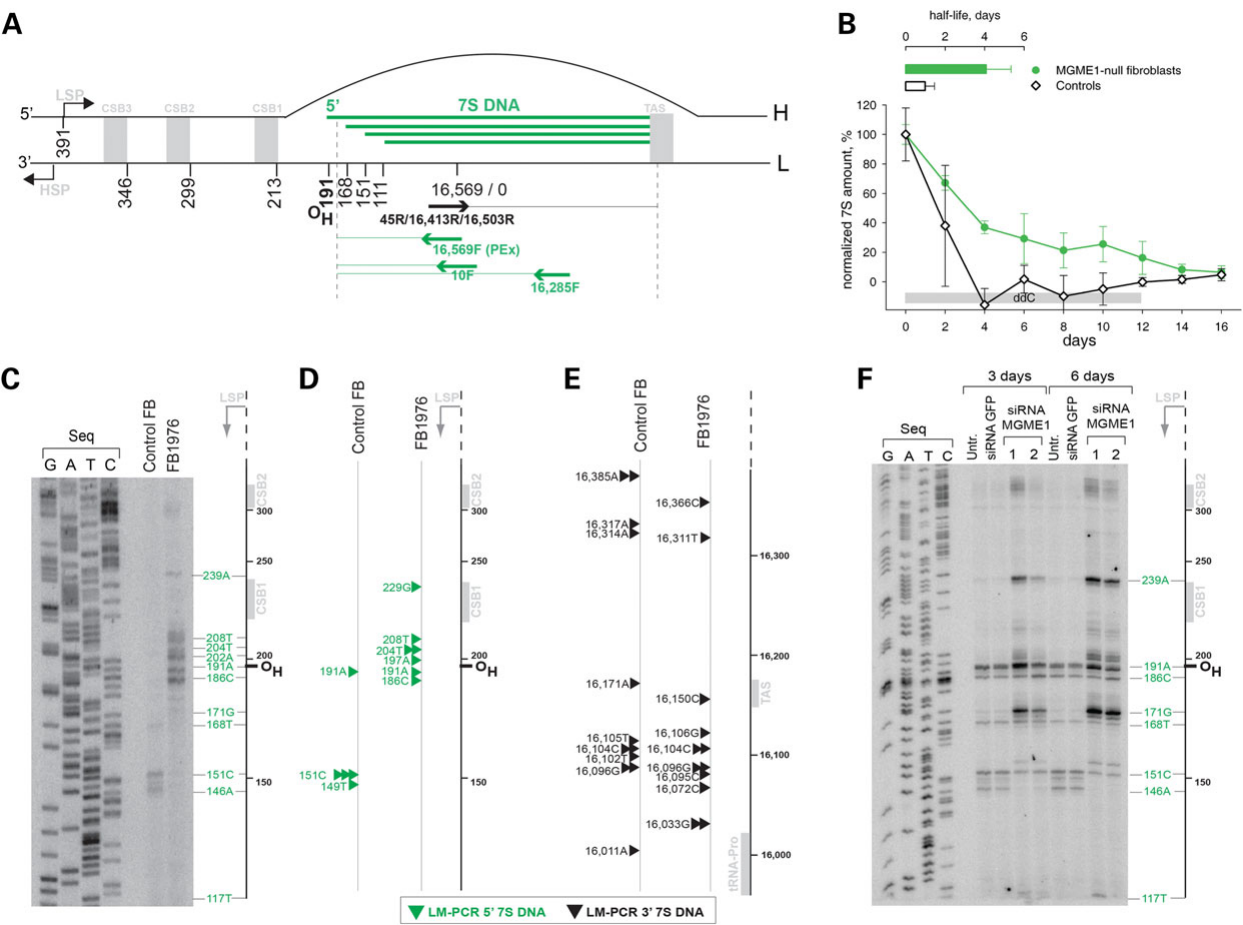
Loss of MGME1 modifies the 5′ ends of DNA species in the NCR. (**A**) The human mitochondrial NCR. The position of the main 5′ ends of 7S DNA found in this and other studies (6–8) are indicated. HSP and LSP—heavy- and light-strand promoter, respectively. CSB—conserved sequence block, TAS—termination associated sequences. Positions of primers used in the PEx and LM-PCR analysis are given. (**B**) 7S DNA levels in control and patient fibroblasts quantified by qPCR during ddC treatment for the indicated time (grey bar). Two controls and two patient samples were investigated, each determined in three independent reactions. Half-life times were determined by nonlinear regression analysis of qPCR data assuming simple exponential decay kinetics (inset). (**C**) Primer extension mapping of DNA 5′ ends in human control and patient fibroblasts (FB1976). A radioactively labelled primer annealing at nt position 16 569–18 of human mtDNA was extended using total DNA preparations treated with RNase A during cell lysis. (**D**) DNA 5′ ends detected by LM-PCR in controls and FB1976. Further details are provided in Supplementary Material, Figure S2. (**E**) DNA 3′ ends in the vicinity of the TAS sequence detected by LM-PCR in human control and patient fibroblast (FB1976). (**F**) PEx mapping of DNA 5′ ends in the human mtDNA NCR of HeLa cells transfected with siRNA to MGME1 or siRNA to GFP for 3 or 6 days. The experiment was performed as per (C). ‘Untr’—non-transfected control.

It has been proposed that mammalian mitochondria operate multiple mechanisms of replication, and each of these models would benefit from further corroboration (1). The initially proposed model (the ‘strand-displacement model’) assumes that replication of mitochondrial DNA (mtDNA) involves only two priming events (one per strand), using a single replicase, Polγ (a holoenzyme composed of the catalytic subunit PolgA and accessory subunit PolgB). Replication of the heavy strand initiates at a specific site in the mtDNA non-coding region (NCR) known as O_H_. DNA synthesis is continuous and proceeds by displacing approximately two-thirds of the length of mtDNA, before O_L_ is exposed and continuous light-strand synthesis is initiated (2). According to this model, the displaced H-strand is proposed to be coated with ssDNA-binding protein (mtSSB). It was later proposed that RNA, rather than protein, is used to coat the displaced H-strand, and this is known as the ‘RITOLS model’ (3,4). In addition, thorough analysis of mtDNA replication intermediates by two-dimensional agarose gel electrophoresis has revealed structures typical of conventional bidirectional replication (5). The NCR of many mtDNA molecules contains a short displacement loop region (D-loop), formed by the presence of a third DNA strand known as 7S DNA. 7S DNA synthesis is believed to be primed by a transcript originating from the light-strand promoter (LSP), which undergoes a transition from RNA to DNA synthesis downstream from LSP. The main 5′ ends of 7S DNA coincide with O_H_, and so this ssDNA species is often considered as a product of prematurely terminated replication initiated at O_H_ (Fig. 1A). However, the exact role of 7S DNA is unknown (9).

Genetic diseases that are associated with failures of mtDNA replication, collectively known as mitochondrial DNA maintenance disorders, have helped to provide deeper insights into the mechanism of mtDNA replication. The main features of these disorders are either decreased mtDNA copy number (depletion) or the presence of multiple mtDNA deletions, and very often a combination of both (10). This group of diseases is caused by mutations in nuclear genes that code for proteins involved in mitochondrial genome maintenance. Pathogenic mutations have been described either in genes that are essential for mtDNA synthesis, namely *POLG* (11), *POLG2* (12), *C10orf2* (13) and possibly *DNA2* (14) or in those involved in the mitochondrial biosynthesis pathways of deoxyribonucleoside-5′-triphosphates (15–20). For some mtDNA maintenance disorders, the pathomechanism has yet to be fully determined (21–24). Recently, we discovered that in addition to the protein products of *POLG*, *POLG2* and *C10orf2*, a highly conserved mitochondrial protein with exonuclease activity, MGME1 (also known as Ddk1 or C20orf72), is required for proper mtDNA replication (25). Loss of function mutations in MGME1, which abolish expression of the protein in patient cells, resulted in the apparent accumulation of unusually large deletions, a reduction of mtDNA copy number and the accumulation of replication intermediates and of 7S DNA. We showed that MGME1 belongs to the PD-(D/E)XK nuclease superfamily and is a close homologue of the bacterial RecB nuclease involved in DNA recombination. We also provided evidence that MGME1 preferentially degrades ssDNA (over dsDNA) in both 5′ → 3′ and 3′ → 5′ directions and is capable of processing DNA flap and RNA–DNA flap intermediates formed during mtDNA synthesis (25) and is involved in the maintenance of proper 7S DNA levels (25,26). The ability of MGME1 to process RNA primers from flap structures resembling replication intermediates is directly relevant to all current models of mtDNA replication. The observation that the levels of 7S DNA are modified by changes in MGME1 activity may be a further important contribution to this puzzle.

In this work, we find that loss of MGME1 activity leads to (i) lengthening of 7S DNA owing to incomplete processing of 5′ ends, (ii) the accumulation of an 11-kb linear mtDNA fragment, a by-product of mtDNA replication originating from chromosome breakage at fragile sites and (iii) accumulation of unusual mtDNA rearrangements (mostly partial duplications). Homologous recombination-dependent DNA repair has been proposed to be involved in the formation of classical deletions in the mtDNA major arc (27). In MGME1-deficient cells, however, only atypical clusters of breakpoints of rearranged mtDNA molecules occur, suggesting that homologous recombination is impaired in these cells. Finally, we show that MGME1 interacts with the mitochondrial replicase PolgA, indicating that it is an additional constituent of the mitochondrial replisome that provides it with a previously not described exonucleolytic activity.

## RESULTS

### Modified DNA 5′ termini in the mtDNA non-coding region of MGME1-deficient cells

Previously, we observed increased levels of 7S DNA in muscle tissues and fibroblasts from patients harbouring a homozygous stop mutation in the *MGME1* gene (25) as well as in cells subjected to siRNA down-regulation of MGME1 (25,26). Given that MGME1 is a nuclease, this result suggested that the enzyme is involved in the turnover of this molecule. In order to verify this hypothesis, we compared the half-lives of 7S DNA in fibroblasts from MGME1-mutated patients and controls upon inhibition of DNA synthesis using dideoxycytidine (ddC) (Fig. 1B). We observed a reduced rate of 7S DNA decay in MGME1-null fibroblasts in comparison with controls (Fig. 1B and Supplementary Material, Table S1). Next, 7S DNA decay was measured in control and MGME1-null fibroblasts upon addition of ethidium bromide (EtBr). EtBr rapidly inhibits mitochondrial transcription that is required for initial steps of 7S biosynthesis (Fig. 1A). EtBr treatment led to a fast 7S DNA decay in control cells; however, the degradation rate was markedly reduced in MGME1-null fibroblasts (Supplementary Material, Fig. S1). This suggests that the degradation of 7S DNA, rather than its synthesis, is impaired in MGME1-deficient cells.

We hypothesized that if 7S DNA is a substrate of MGME1, then a deficiency of MGME1 should result in altered processing of 7S DNA. To verify this, we performed primer extension (PEx) analysis of DNA 5′ ends in the NCR of MGME1-null patient fibroblasts (FB1976) (Fig. 1C). All DNA samples were treated with RNase A prior to PEx to remove potential 5′ RNA leaders. The PEx primer annealed at the NCR downstream from previously identified DNA 5′ ends (mtDNA positions 16 569–18) (6) and was extended towards LSP (Fig. 1A). PEx analysis of DNA from the P1976 fibroblasts showed more abundant extended species with 5′ ends upstream from the previously observed ends (Fig. 1C), consistent with defects in the processing of 5′ termini of 7S DNA. The presence of elongated 7S DNA 5′ ends was confirmed by ligation-mediated (LM) PCR (Fig. 1D, arrowheads and Supplementary Material, Fig. S2A). In addition, we used LM-PCR to investigate 3′ ends of 7S DNA. We found no changes at the 3′ end of 7S DNA upon loss of MGME1 activity in comparison with controls (Fig. 1E), consistent with the ability of MGME1 to catalyse an 5′ → 3′ exonucleolytic reaction as reported previously (25,26).

Under the assumption that the synthesis of 7S DNA is initiated by transcription from LSP followed by an RNA–DNA transition downstream from LSP, we examined whether inactivation of MGME1 affects the processing of RNA at the 5′ end of 7S DNA. To this end, we performed PEx on DNA samples that were not treated with RNase A during the isolation procedure (Supplementary Material, Fig. S3). The DNA polymerase used for PEx can perform reverse transcription of an RNA template if primed on a DNA template, so any RNA covalently attached to the 5′ ends of DNA should also be extended (Supplementary Material, Fig. S3A). We did not detect any extra ends in P1976 fibroblast samples that were not treated with RNase A compared with those present in the same samples treated with RNase A (Fig. 1C and Supplementary Material, Fig. S3B). Furthermore, pre-treatment of ﬁbroblast DNA with RNase H1 (which specifically degrades the RNA strand of RNA–DNA hybrids) prior to PEx produced an extra minor band at CSB2 in both control ﬁbroblasts and FB1976 (Supplementary Material, Fig. S3B). This species has been previously observed after RNase H1 treatment of mtDNA (6,7) and is believed to represent transcription events initiating from LSP and terminating at CSB2. These results suggest that the RNA–DNA transition is not affected in MGME1-null cells and that the enzyme takes part in DNA processing downstream from the transition site.

In order to see whether partial inactivation of MGME1 expression also has an effect on the processing of 7S DNA, we performed siRNA silencing of MGME1 in HeLa cells followed by analogous PEx examination of DNA 5′ ends in the NCR region. MGME1 protein levels were efficiently reduced by the siRNA treatment, being undetectable by western blotting following 6 days of transfection (Supplementary Material, Fig. S3C). This analysis confirmed the existence of the previously observed DNA 5′ ends in the NCR, as well as revealing more abundant species with 5′ ends upstream from the known termini in MGME1 siRNA-transfected cells (Fig. 1F).

### Overexpression of MGME1 reduces the steady-state levels of 7S DNA and shortens 7S DNA 5′ ends

**Figure 2.**
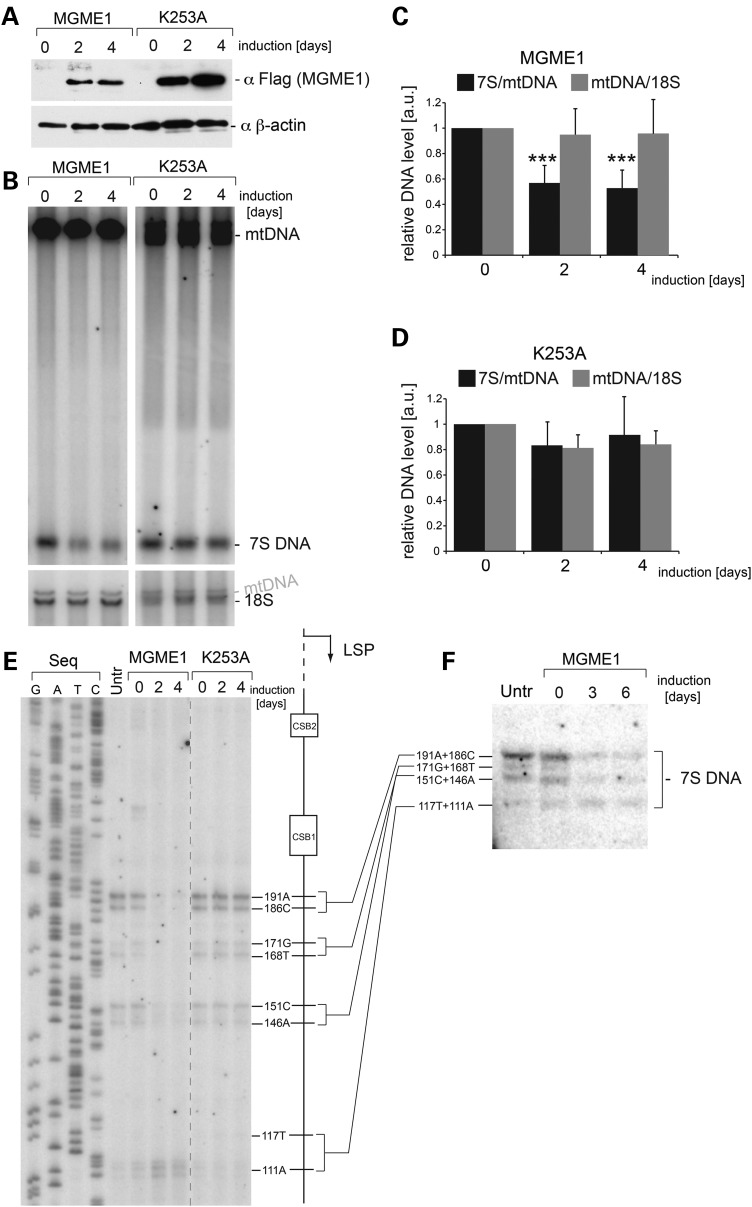
Overexpression of MGME1 modifies the steady-state levels and length of 7S DNA. (**A**) Western blot of a time course of expression of MGME1.Flag.Strep2 or the K253A mutant in HEK293T cells. The MGME1 protein was detected using anti-Flag antibodies; β-actin used as a loading control. (**B**) Total DNA from cells expressing MGME1 wild-type or the K253A catalytic mutant in a 4-day time course was analysed by 1D Southern blotting with a probe specific for the NCR (mtDNA region: 14 258–4121). 18S rDNA was used as a loading control. (**C**) and (**D**) Quantification of the Southern blots as per (A) for MGME1 wild-type (B) or K253A (C). The values of the relative DNA level (7S DNA/mtDNA—black or mtDNA/18S rDNA—grey) were normalized for the values obtained for uninduced cells. ***P < 0.001; two-tailed Student's *t*-test; *n* = 7 for MGME1 wt, *n* = 3 for K253A, error bars = 1 SD. (**E**) Mapping of DNA 5′ ends in the human mtDNA NCR of HEK293T cells expressing MGME1 wild-type or K253A in a 4-day time course. PEx was performed as per Figure 1C. (**F**) Total DNA from cells expressing MGME1 wild-type in a 6-day time course was analysed using a high-resolution (4% denaturing polyacrylamide gel) 1D Southern blot with a probe as per (A). The 7S DNA species containing the DNA 5′ ends mapped by PEx in (D) are indicated. ‘Untr’—non-transfected control.

In order to establish whether elevated levels of MGME1 affect mtDNA metabolism, and in particular the level and length of 7S DNA, we overexpressed recombinant MGME1 or the K253A catalytic mutant (both tagged with Strep2 and Flag) (25) in HEK293T cells in a time course of 4 days (Fig. 2A). In these conditions, overexpression of either the wild-type or mutant MGME1 in HEK293T cells did not have any significant effect on mtDNA copy number (Fig. 2B–D). This result is inconsistent with our previous report, where high overexpression levels through a lentiviral system in human primary fibroblast led to reduced mtDNA copy numbers (25). As the overexpression levels are comparable in both cell lines (Supplementary Material, Fig. S4A), this effect is likely owing to different cell types used and differences in the duration of overexpression (days in the case of HEK293T cells, as opposed to several weeks in the case of human fibroblasts). Despite unaltered mtDNA copy numbers, there was an ∼50% reduction in the steady-state level of 7S DNA in HEK293T cells overexpressing the wild-type version of MGME1 (Fig. 2B, left and C), but not in cells overexpressing the K253A mutant (Fig. 2B, right and D). An extended time course of MGME1 overexpression did not cause any more pronounced effect upon 7S DNA steady-state levels. However, we have noticed that these DNA species appeared shorter in the MGME1 wild-type overexpressing cells (Supplementary Material, Fig. S4B).

In order to test whether an overabundance of MGME1 also causes shortening of 7S DNA owing to altered processing of 5′ ends, we performed PEx analysis within the NCR, as described in the preceding section. This PEx analysis showed modified 5′ ends in DNA samples from HEK293T cells overexpressing wild-type MGME1, but not the K253A mutant. The intensity of PEx bands corresponding to DNA ends upstream of position 111 was notably lower upon MGME1 wild-type overexpression (Fig. 2E), consistent with the observed shortening observed in Southern blotting analysis of the same samples (Supplementary Material, Fig. S4B). An extended time course of MGME1 overexpression did not cause any more pronounced effect on 7S DNA ends (Supplementary Material, Fig. S4C). The changes in 7S DNA upon overexpression of wild-type MGME1 were further documented by high-resolution Southern blotting (Fig. 2F). Only the longest 7S DNA molecules, with 5′ ends upstream from nt 117, were modified after overexpression of MGME1 wild-type, consistent with the results obtained in PEx analysis (compare Fig. 2E and F). In summary, these results support the proposed role of MGME1 in regulating the proper steady-state levels of 7S DNA (25,26) and point towards its function in the processing of the 5′ terminus of this molecule.

### *In vitro* activity of MGME1 on D-loop DNA substrates

As the above-mentioned data suggested that MGME1 operates at the 5′ terminus of the mitochondrial D-loop region, we have investigated the *in vitro* enzymatic properties of MGME1 on D-loop-type DNA substrates. The recombinant protein was isolated from human mitochondria by affinity purification as described previously (25). As reported earlier, MGME1 efficiently cleaves ssDNA (Supplementary Material, Fig. S5A, first panel), but not dsDNA, and requires DNA ends in order to initiate the reaction (25). We additionally tested whether the enzyme is capable of cleaving displaced ssDNA in a DNA bubble structure (Supplementary Material, Fig. S5A, second panel). However, recombinant MGME1 processed this substrate with low efficiency (Supplementary Material, Fig. S5B, orange). Next, we tested whether MGME1 can process a D-loop strand (a linear fragment that displaces one strand of dsDNA) in a D-loop structure; such a strand would correspond to 7S DNA in the mitochondrial NCR (Supplementary Material, Fig. S5A, four right panels). The recombinant MGME1 was not able to process a D-loop strand that was fully hybridized within the structure (Supplementary Material, Fig. S5B, green). However, when the D-loop strand formed a flap protruding outside of the displaced DNA region, MGME1 was able to cleave the flap with reasonable efficacy (Supplementary Material, Fig. S5B, red). A point mutation of the conserved Lys residue of the PD-(D/E)XK domain completely abolished all nucleolytic activities tested (Supplementary Material, Fig. S5A, K253A). These results suggest that effective processing of 7S DNA by MGME1 in mitochondria would require partial unwinding of the D-loop region, so that flap structures are generated at the 5′ ends of 7S DNA, making them accessible for the enzyme.

### Loss of MGME1 results in replication-dependent chromosomal breakage of mtDNA

Pathogenic mutations that cause mtDNA replication defects often lead to deletions and rearrangements of the mitochondrial genome (10). Much less is known about the role of linear fragments of mtDNA in pathogenesis. Chromosomal breakage of mtDNA near O_H_ and O_L_, manifested by the presence of a linear ∼11-kb double-stranded mtDNA fragment corresponding to the major arc, has been associated with replication pausing in a mouse model expressing a proofreading-deficient mutant of the mitochondrial DNA polymerase gamma [Polγ, ‘mutator mouse’, (28,29)]. It has been proposed that these linear sub-genomic fragments are generated by spontaneous nicking of single-stranded DNA at replication forks arrested near O_H_ and O_L_. Such fragments can also be generated by digestion of normal mtDNA samples with S1 nuclease, as the junctions near O_H_ and O_L_ are typically susceptible to nicking (29).

**Figure 3.**
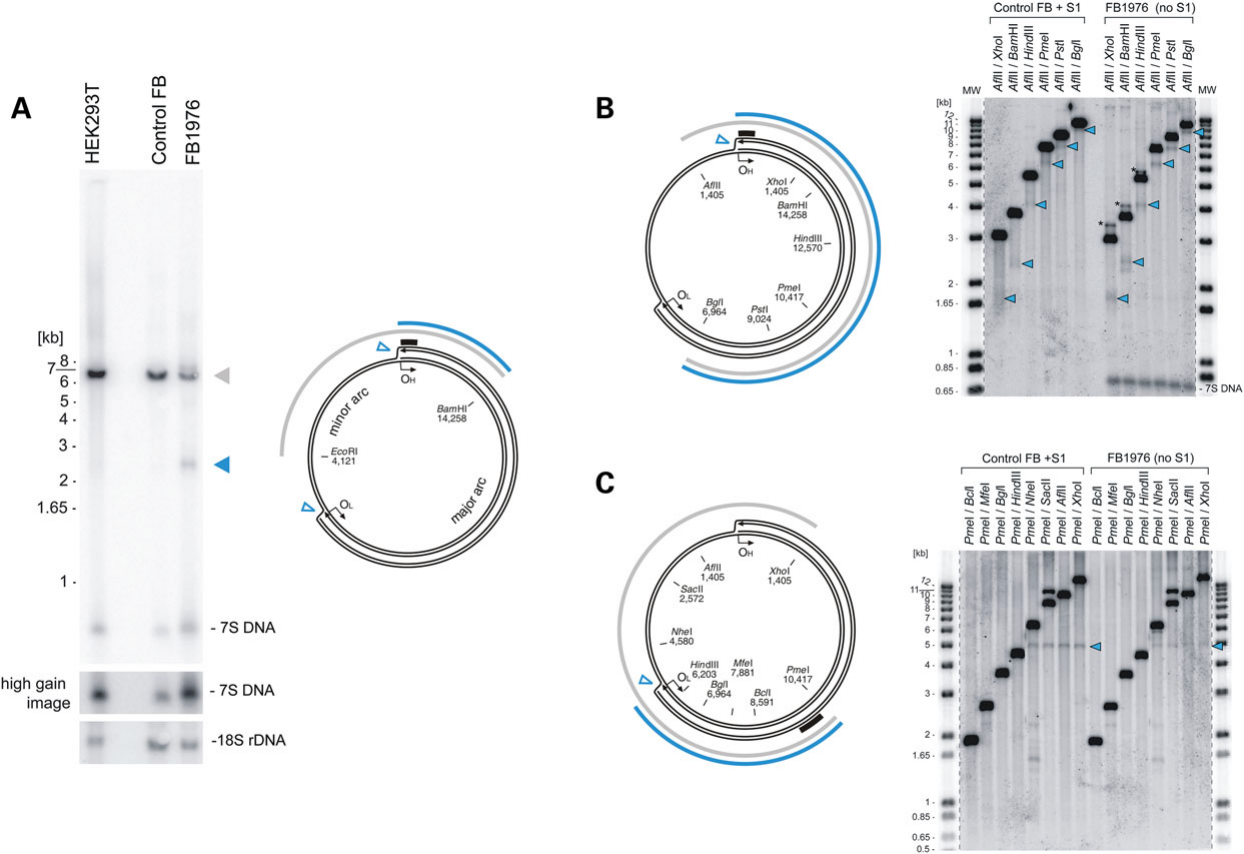
Loss of MGME1 results in accumulation of an 11-kb sub-genomic mtDNA fragment. (**A**) (left) Total DNA from HEK293T cells, control and patient fibroblasts (FB1976, cell passage 8) was digested with *Bam*HI and *Eco*RI and analysed by 1D Southern blot with a probe specific for the NCR (mtDNA region: 16 341–151). 18S rDNA was used as a loading control. Blue arrowhead—2.5-kb sub-1n fragment observed recurrently for FB1976. (right) Schematic representation of the source of the sub-genomic fragment. Grey arc—the full-length genomic fragment. Empty blue arrowheads—chromosomal breakage of replication intermediates near O_H_ and O_L_. Blue arc—2.5-kb sub-1n fragment. Black bar—the probe. (**B**) (left) The restriction map of human mtDNA indicating sites relevant for the analysis. Blue arc—the sub-genomic fragment near O_H_. Black bar—the probe (mtDNA region: 16 341–151). (right) Total DNA from control fibroblasts treated with S1 nuclease or from FB1976 was analysed by 1D Southern blotting using indicated restrictases. Blue arrowheads—the sub-genomic fragments generated by S1 nuclease treatment of control cells or present in FB1976. Asterisks indicate the main genomic restriction fragment with residual 7S DNA still bound (Supplementary Material, Fig. S5B). (**C**) (left) The restriction mapping of the sub-genomic fragments near O_L_ (blue arc). Black bar—the probe (mtDNA region: 9 610–10 218). (right) 1D Southern blot analysis as per (B) using restriction enzymes as indicated. The remaining indications as per (B).

We examined mtDNA from MGME1-null patient fibroblasts by Southern blotting, and in addition to the previously reported reduction in mtDNA copy number, we also observed an mtDNA fragment below the expected restriction size (sub-genomic fragment) (Fig. 3A). The size of this band was in agreement with a potential chromosomal breakage near O_H_ observed previously in the mutator mouse (28,29). In order to map both ends of the sub-genomic fragment, more detailed restriction mapping followed by detection of mtDNA by Southern blotting was performed in FB1976, using S1-treated DNA of normal fibroblasts as a control (Fig. 3B and C). First, mtDNA was digested using an enzyme with a recognition site upstream from O_H_ (Fig. 3B, *Afl*II) in combination with a series of enzymes that cut downstream of O_H_ in the mtDNA major arc (Fig. 3B, *Xho*I, *Bam*HI, *Hin*dIII, *Pme*I, *Pst*I, *Bgl*II). For each enzyme combination, a radioactive probe that binds within the predicted sub-genomic fragment (Fig. 3B, black bar) detected a series of fragments consistently smaller than the genomic restriction enzyme fragment by ∼1.2–1.3 kb (Fig. 3B, blue arrowheads). On the other hand, a radioactive probe that binds upstream of O_H_ and outside of the predicted sub-genomic fragment (Supplementary Material, Fig. S6A, black bar) did not detect any sub-genomic bands in S1-nuclease-treated samples nor in the MGME1-null patient fibroblasts. One end of the sub-genomic fragment is therefore located in the vicinity of O_H_.

In order to determine the site of the opposite end of the sub-genomic fragment, we performed restriction mapping using an enzyme that cleaves in the mtDNA major arc (Fig. 3C, *Pme*I) in combination with a series of enzymes that cut in the mtDNA major and minor arc (Fig. 3C, *Bcl*I, *Mfe*I, *Bgl*I, *Hin*dIII, *Nhe*I, *Sac*II, *Afl*II and *Xho*I) and a radioactive probe binding in the major arc. The sub-genomic fragment was detected in FB1976 and S1-treated fibroblasts only when a restriction enzyme cut within the minor arc (distal to O_L_) was used. This localizes the end of the fragment close to O_L_ (i.e. between the *Hin*dIII and *Nhe*I sites, Fig. 3C, right). Together these results indicate that the ends of the sub-genomic species detected in the MGME1-null patient fibroblast map near O_H_ and O_L_, and therefore, it consists of the entire mtDNA major arc.

**Figure 4.**
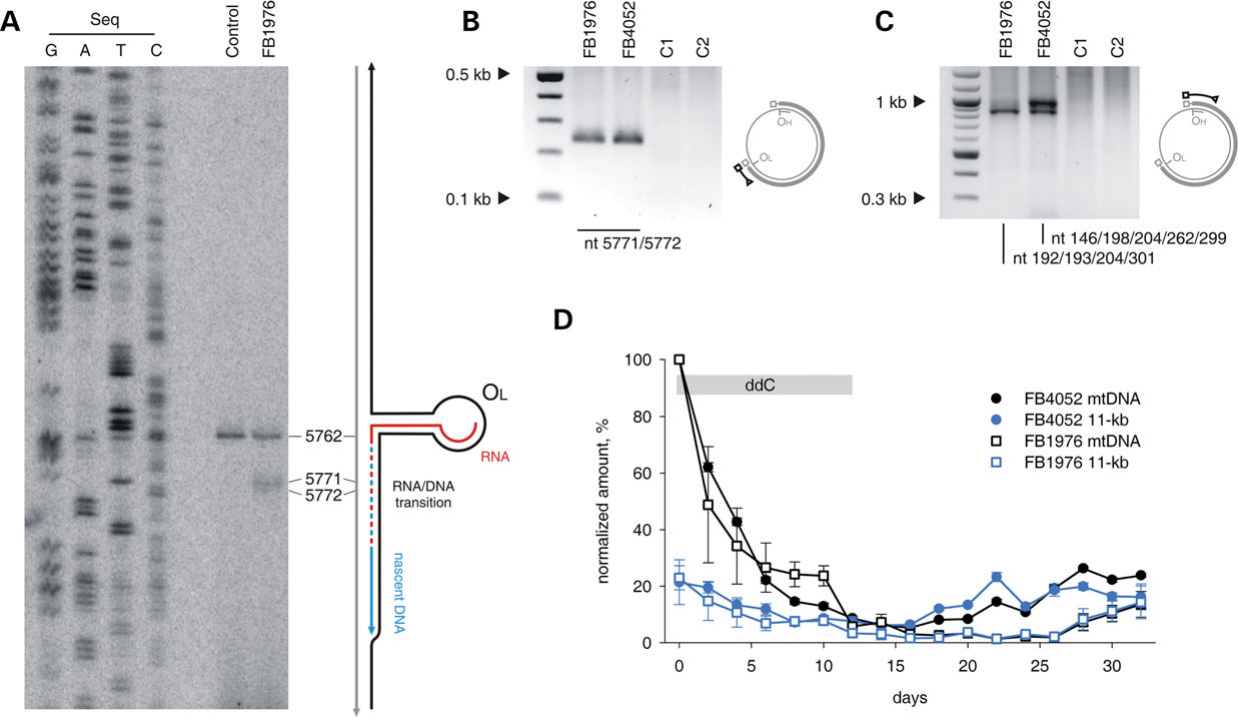
Blunt ends and prolonged persistence of the 11-kb sub-genomic mtDNA fragment in MGME1-deficient fibroblasts. (**A**) PEx mapping of DNA 5′ ends near O_L_ in control and FB1976. A radioactively labelled primer annealing at nt position 5915–5898 of human mtDNA was extended using total DNA preparations treated with RNase A. Right: the predicted hairpin structure at O_L_ and the current model of O_L_-specific initiation of lagging-strand mtDNA synthesis (30). (**B**) Double-stranded blunt ends close to O_L_ in patient fibroblasts detected by LM-PCR. Amplification was performed using a linker-specific and an mtDNA-specific primer. The ends were identified by sequencing 12 single-molecule amplicons. C1, fibroblasts from an 11-year-old healthy control; C2, fibroblasts from a 39-year-old healthy control. (**C**) Double-stranded blunt ends in the vicinity of O_H_. Note that while 7S DNA 5′ ends are present in this region both in patients and in controls (Fig. 1**F**, Supplementary Material, Fig. S2), blunt dsDNA ends are specific for MGME1-deficient patients. Positions of mapped ends are indicated. (**D**) The presence of the 11-kb sub-genomic mtDNA fragment in MGME1-deficient fibroblasts mtDNA during depletion-repopulation experiment. Fibroblasts from patients were treated with ddC for 12 days (indicated by grey bar) followed by culturing without the inhibitor for 20 days. Genomic (black) and sub-genomic (blue) fragment amounts were quantified by qPCR and normalized to copy numbers of complete ds mtDNA molecules measured at the beginning of the ddC treatment. Error bars—SD values. *n* = 3.

The exact nucleotide positions of the DNA ends of the sub-genomic fragment near O_L_ have been determined by PEx and LM-PCR (Fig. 4A–C). The PEx analysis of O_L_ ends in RNase A-treated DNA samples revealed a strong pause at position 5762 in both control and patient fibroblasts, likely owing to a hairpin secondary structure at O_L_ reported previously (30). Additional DNA 5′ ends downstream from the predicted O_L_ hairpin structure were detected in the FB1976 MGME1-null fibroblasts that were not seen in control fibroblasts. These additional DNA ends mapped to positions 5771 and 5772 (Fig. 4A). LM-PCR amplification after ligation of a blunt-ended dsDNA linker to free dsDNA ends revealed that positions 5771 and 5772 not only mark the 5′ end of an L-strand fragment but that they represent patient-specific free blunt ends of a double-stranded sub-genomic fragment (Fig. 4B, Supplementary Material, Fig. S2C). In contrast to the PEx analysis, the LM-PCR approach did not show any free ends in the immediate vicinity of the O_L_ hairpin in these conditions (position 5762), which corroborates the assertion that the PEx signal detected at this position results from polymerase pausing at a strong secondary structure and does not represent true ends.

Linker-ligated DNA samples were also examined by using an mtDNA-specific primer located close to the O_H_ end of the sub-genomic fragment, but outside of the 7S DNA region. This analysis revealed that double-stranded blunt ends were present at positions similar to those detected by PEx or LM-PCR using primers within the 7S region (Fig. 1C and D). While 5′ 7S DNA ends close to O_H_ were also present in controls, the sub-genomic fragment O_H_ ends were specific for the MGME1-deficient patients and were not detected in controls (Fig. 4C).

Next, we investigated changes in the amount of the 11-kb sub-genomic fragment in conditions of different mtDNA replication activity (Fig. 4D). To this end, sub-genomic fragment levels were measured using qPCR comparing the major arc (sub-genomic + genomic) and the minor arc (genomic) during replication inhibition and subsequent mtDNA repopulation. In MGME1-null fibroblasts, in accordance with measurements by Southern blotting, ∼20% of FB1976 mtDNA was present as sub-genomic fragments before ddC treatment. Similarly to full-length mtDNA, the amount of the sub-genomic fragment decreased during replication inhibition, although at slower kinetics (Supplementary Material, Table S1). During recovery, the amount of sub-genomic mtDNA (Fig. 4D, blue traces) increased parallel to total mtDNA copy numbers (Fig. 4D, black traces). These findings suggest that the turnover of the 11-kb sub-genomic fragment is delayed in MGME1-deficient cells and it is *de novo* generated during mtDNA repopulation.

### Loss of function mutations in MGME1 results in unusual large-scale mtDNA rearrangements

**Figure 5.**
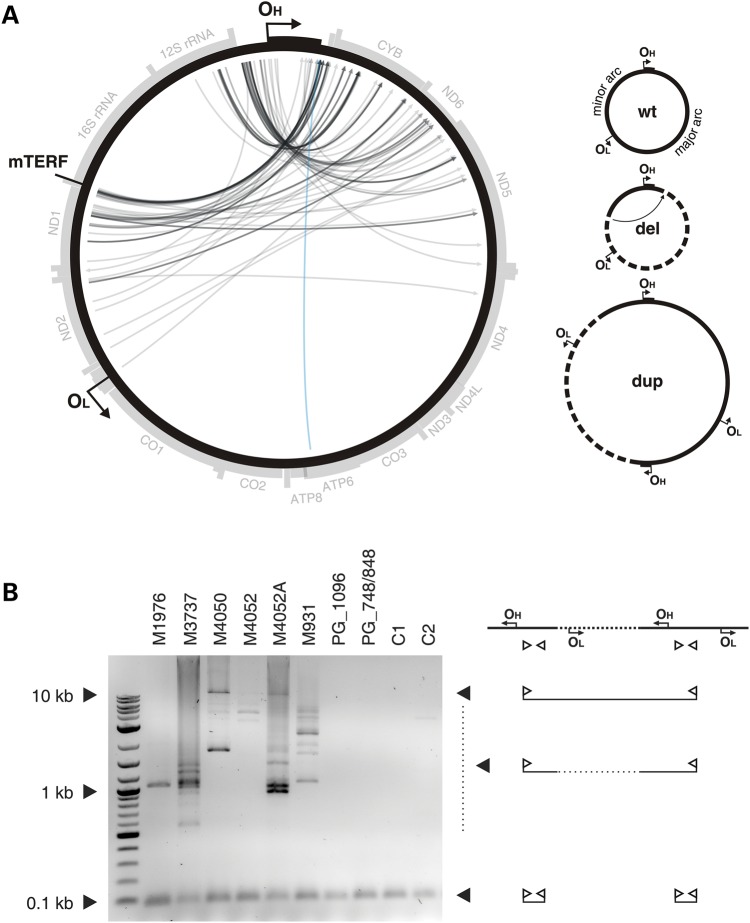
Unusual mtDNA rearrangements in *MGME1* patients. (**A**) Circos representation of detected mtDNA breakpoints in patients with pathogenic MGME1 mutations. Arrowheads indicate the orientation of the deletions. The part of the genome that is deleted spans counter clockwise from the root to the head of the arrow (as indicated by dashed line in the panel ‘del’ of the scheme on the right). A detailed list of the detected breakpoints is available in Supplementary Material, Table S2. Breakpoints that conform to regular major arc deletions are marked in blue; grey arcs indicate breakpoints that remove O_L_. Possible interpretations of the breakpoints are shown in the scheme on the right. del, deleted mtDNA molecules; dup, partially duplicated mtDNA; wt, wild-type. (**B**) Partially duplicated mtDNA molecules in skeletal muscle of *MGME1* patients as detected by long-extension PCR. The scheme on the right indicates the interpretation of the detected bands. Bands at 0.1 kb represent short PCR fragments deriving from primers annealing at neighbouring positions. Bands at 0.5 kb and above originate from partial or complete duplications of mtDNA. PG_1096, 6-year-old patient with homozygous mutation R1096C in POLG; PG_748, 12-year-old patient with compound heterozygous mutations W748S and G848S in POLG; C1, 35-year-old healthy control; C2, 63-year-old healthy control.

Previously, we reported that patients with defective MGME1 accumulate deletions of mtDNA in affected tissues (25). The amount of apparently deleted molecules was comparable with levels found in patients with pathogenic *POLG* mutations and ranged from 0.04 to 1.5%. In contrast to POLG patients, however, breakpoints that were consistent with unusually large deletions spanning almost two-thirds of the mitochondrial genome were present in *MGME1* patients. To better understand the nature of these rearranged mtDNA molecules in MGME1-null cells, we determined exact mtDNA breakpoints by single-molecule amplification and subsequent sequencing (Fig. 5A, Supplementary Material, Table S2). For comparison, the spectrum of breakpoints detected in a patient with mutated *POLG* is shown in Supplementary Material, Figure S7 and Table S3. Out of 131 detected breakpoints, only in a single case, both breakpoints were located within the major arc (Fig. 5A, blue arrow), where most mtDNA deletions described in the literature are situated, and which is also typical for mutated *POLG* (Supplementary Material, Fig. S7 and Table S3). The end positions of the other breakpoints (Fig. 5A, grey arrows) were similar to those reported for other pathological conditions and were mainly located between positions nt 13 000 and nt 16 100 in the major arc. However, starting positions were located within the minor arc and clustered either between O_H_ and the heavy strand promoter (HSP), in the 12S ribosomal RNA gene or downstream of the tRNA^LeuUUR^ gene, which is also a binding site for the mitochondrial transcription termination factor mTERF1 (31). The vast majority of breakpoints would be consistent with deletions eliminating more than two-thirds of the mitochondrial genome (Fig. 5A, grey arrows) and would remove the O_L_ replication origin. This is notable, because it has been claimed that O_L_ is essential for mtDNA maintenance, and so molecules lacking O_L_ should be unable to propagate (32). However, we observed clear replicative clonal expansion of many mtDNA species with unusual breakpoints, because they were individual-specific and were detected many times in a single individual (Supplementary Material, Table S2). To address the paradox of the clonal expansion of apparently replication-incompetent molecules, we determined whether breakpoints in the *MGME1* patients could represent partial duplications instead of true deletions. Investigation by long-extension PCR (Fig. 5B) showed that, in addition to a short PCR product, large products were also detectable in skeletal muscle samples of *MGME1* patients. This indicates that the short primer-binding region was present at least twice on the same mtDNA molecule, which is a hallmark of duplications and multiplications (Fig. 5B). Similar large products were not detected either in control skeletal muscle samples, or in samples from POLG patients, despite the fact that the latter contain an increased number of regular deletions (cf. Supplementary Material, Fig. S7 and Table S3).

Furthermore, we observed that some of the breakpoints were associated with complex rearrangements within the NCR. In these rearranged molecules, one or two short tandem duplications of the NCR were additionally inserted between the long-distance breakpoints (Supplementary Material, Table S1). Similar tandem duplications of the NCR were previously reported in humans (33) and in a mouse model with proofreading-deficient Polγ (34), although these were of larger size. While duplications were found to be 150–880 base pairs long in these previous studies, we observed insertion lengths of 13–287 base pairs in skeletal muscle of MGME1-deficient patients. In each complex rearrangement, at least one breakpoint was located in a well-defined region between nucleotides 16 069 and 16 084. An accumulation of deletion breakpoints in this specific region in the immediate vicinity of the 3′ end of the 7S DNA has been reported in a mouse model with elevated rates of double-strand breaks (35).

### MGME1 interacts with PolgA, but not other mitochondrial nucleoid proteins

**Figure 6.**
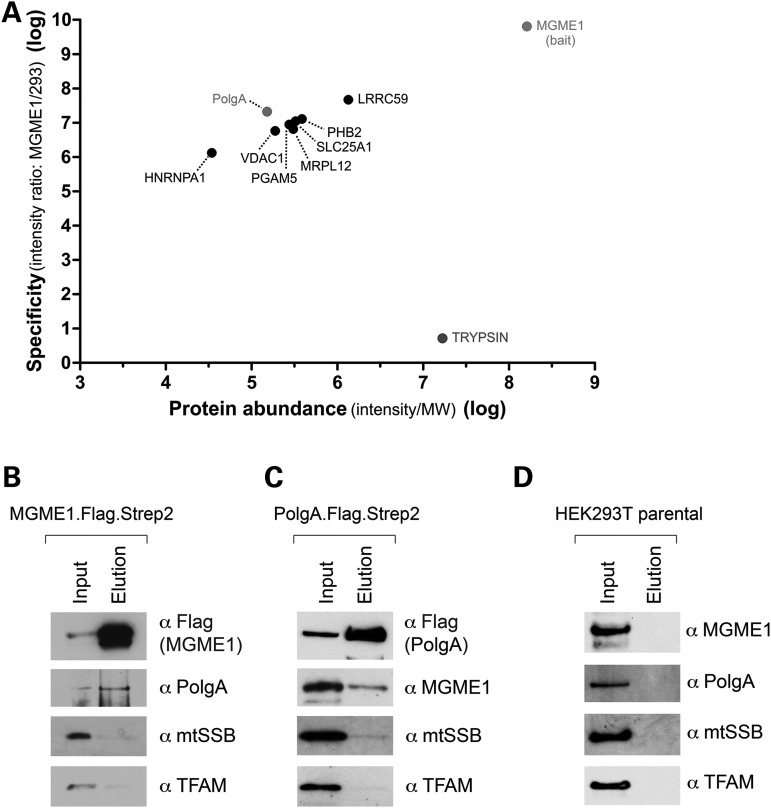
Interaction of MGME1 and PolgA. (**A**) Mitochondria from parental HEK293T cells or derivatives expressing MGME1.Flag were isolated, lysed, and protein extracts were subjected to affinity purification using anti-Flag beads. Graph presents quantitative analysis of MS data obtained on eluates. Protein names are displayed. MGME1, PolgA and trypsin are highlighted. The entire dataset is specified in Supplementary Material, Table S5. (**B**) Western blot result of pull-down of the endogenous PolgA with MGME1.Flag.Strep2. The mitochondrial lysates (‘Input’) and eluates (‘Elution’) were analysed with the antibodies as indicated. (**C**) Reciprocal western blot confirmation of the MGME1–PolgA interaction by co-IP of the endogenous MGME1 with PolgA.Flag.Strep2. (**D**) Western blot of the control experiment performed using parental HEK293T cells, which do not express either recombinant MGME1 or PolgA proteins.

As molecular evidence has suggested that MGME1 is required for proper mtDNA replication, we were interested to see whether MGME1 also physically interacts with proteins known to be important for mtDNA metabolism. In order to address this, a FLAG-tagged version of the protein was introduced to HEK293T cells, and the FLAG tag was used to purify MGME1 and potential interacting proteins from isolated mitochondria. Five bait purifications and one control purification (from an untransfected parental HEK293T cell line) were performed. Co-purified proteins were identified by high-resolution mass spectrometry (MS) and quantified using a label-free approach [see Supplementary Experimental Procedures for details (36,37)]. Protein abundance was calculated as MS signal intensity divided by the molecular mass of the protein. Specificity was defined as the ratio of the mean signal intensity measured in the bait purifications to the intensity determined for the negative control purification. A complete list of identified proteins in all 6 samples consisted of 256 proteins, which were ranked to produce the final list (Supplementary Material, Table S5). Components of cytoplasmic ribosomes and proteins with specificity of <1 were removed from the list. The final list contains proteins which were present in all bait purifications but were absent from the control sample. These criteria led to the list of potential interactors, which were plotted according to specificity and abundance (Fig. 6A). Additionally, we plotted the results for trypsin, which was externally added to the samples during MS analysis (Fig. 6A). Among the potential interactors was PolgA, the catalytic subunit of the mtDNA polymerase (38). Notably, we did not identify other known factors involved in mtDNA transactions, including abundant DNA-interacting proteins of the mitochondrial nucleoid (TFAM and mtSSB) (Fig. 6A and Supplementary Material, Table S5). Therefore, we decided to further study potential PolgA–MGME1 interactions.

The co-purification of PolgA with MGME1 was confirmed by immunoblotting using a FLAG- and Strep2-tagged protein. There was a clear enrichment of PolgA in the MGME1 elution fraction (Fig. 6B). In contrast, there was no appreciable enrichment of known DNA-interacting proteins of the mitochondrial nucleoid (TFAM or mtSSB) (Fig. 6B), confirming that a substantial enrichment of PolgA had been achieved by the affinity purification procedure. In the reciprocal experiment, a tagged version of PolgA carrying FLAG and Strep2 binding groups (PolgA.Flag.Strep2) was expressed in HEK293T cells. PolgA.Flag.Strep2 and interacting proteins were purified on a streptavidin column as described for MGME1.Flag.Strep2 (Fig. 6C). The PolgA elution fraction contained MGME1, and the relative proportion of eluted MGME1 was higher as compared with TFAM or mtSSB. Elution fractions from mitochondrial extracts obtained from parental HEK293T cells, which do not express either of the tagged bait proteins, did not contain any of the investigated proteins (Fig. 6D). These results suggest that a proportion of PolgA interacts with MGME1 in human mitochondria, at least in cells overexpressing these proteins.

## DISCUSSION

### Role of MGME1 in the metabolism of 7S DNA

The processing of the 5′ ends of 7S DNA is not a well-characterized process. Transcription from LSP generates an RNA molecule (7S RNA), which is thought to prime synthesis of 7S DNA (39,40). The conserved sequence blocks (CSBs, Fig. 1A), particularly CSB2 (nt 299–315), have been identified as the main transition sites from RNA to DNA synthesis (6,7,40). However, the major sites of the 5′ ends of 7S DNA map to sites downstream of the CSBs [nt 191 (O_H_), nt 168, nt 151 and nt 110] (6–8) suggesting that the 5′ ends of pre-7S DNA are nucleolytically processed. In our previous work, we reported that loss of MGME1 activity, either through siRNA treatment of cultured cells, or in fibroblasts derived from patients with nonsense mutations in the *MGME1* gene, shows an increase in the steady-state level 7S DNA. Here, we show that abundant longer 7S DNA species can also be detected in these cells, the 5′ ends of which map to immediately upstream of O_H_, (mtDNA nucleotide position 191), as well as specific sites adjacent to Conserved Sequence Block 1 (CSB1) and multiple sites at CSB2 (Fig. 1).

**Figure 7.**
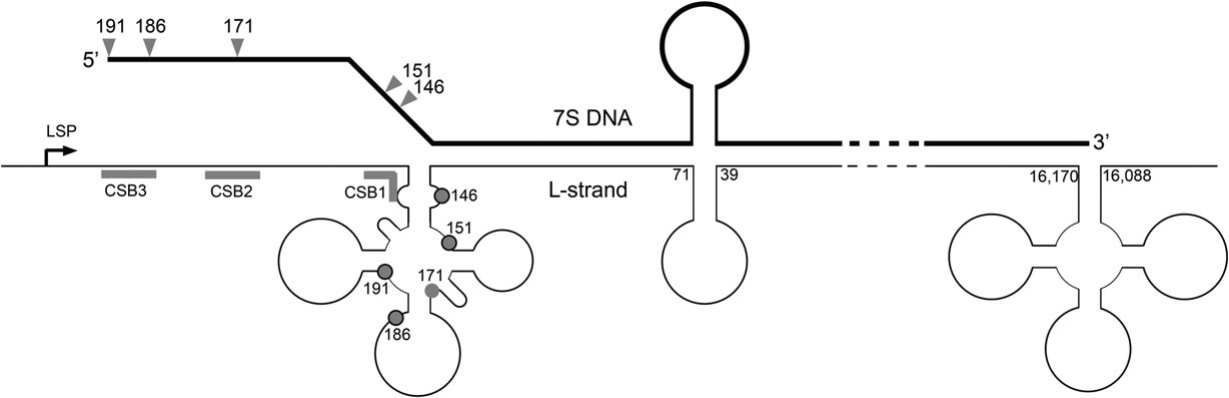
Hypothetical secondary structures in the D-loop region and a role for MGME1. Secondary structures are depicted as forming in the mtDNA NCR region, according to the predictions of Brown *et al*. (41). Formation of a cloverleaf structure in the mtDNA L-strand in the O_H_ region could prevent the 5′ end of 7S DNA from annealing to the template strand, leaving 7S DNA displaced as along 5′ ssDNA ﬂap. The region covered by this cloverleaf structure encompasses almost all of the major sites of 7S DNA 5′ ends. If MGME1 is directly involved in the processing of 7S DNA ends, then the 5′ end of 7S DNA must be displaced into an ssDNA ﬂap in order to be a good substrate for MGME1, according to the *in vitro* characterization presented here. Displacement of the 5′ end of 7S DNA by a secondary structure as shown above could serve this purpose.

In MGME1-deficient cells, the longer 7S molecules could conceivably arise from either (i) aberrant RNA–DNA transitions or (ii) an inability to process the 5′ ends of 7S DNA molecules, leading to an accumulation of longer species. Our experiments indicate that RNA–DNA transitions are not affected in MGME1-null cells (Supplementary Material, Fig. S3), suggesting that the latter possibility is the case. In this scenario, the RNA–DNA transition could occur at CSB2 (as observed previously) or immediately upstream of CSB1 (Fig. 1), followed by MGME1-driven exonucleolytic degradation of the 5′ ends of precursor 7S DNA molecules to the commonly observed downstream sites at O_H_, nt 171 and nt 151. This possibility is also consistent with experiments in which MGME1 was overexpressed in HEK293T cells, where shorter 7S DNA species were observed, which may result from unusually high processing activity in these cells (Fig. 2). The *in vitro* characterization of MGME1 activity has indicated that while 5′ displaced sections of D-loop structures are good substrates for the enzyme, fully annealed D-loop strands cannot be degraded past the dsDNA junction (Supplementary Material, Fig. S5A, right panel). Any precursor 7S DNA would therefore only be a good substrate for MGME1 if its 5′ section is displaced, although whether this is the case *in vivo* has not been studied. However, the sequence at the 5′ end of the D-loop is predicted to fold into a conserved cloverleaf-like secondary structure, incorporating the CSB1 sequence into the stem and encompassing all of the major 5′ ends of 7S DNA observed in control cells from this study (41). If this structure were to form in the template L-strand following D-loop synthesis, then the 5′ end of 7S DNA would be prevented from annealing, forming a large ssDNA flap (Fig. 7). The Twinkle replicative helicase has been reported to be required for 7S DNA synthesis (42), and so it also cannot be excluded that this enzyme is responsible for unwinding the 5′ ends of 7S DNA, making them suitable for MGME1 processing.

It is less clear whether this hypothesis of direct 7S DNA processing by MGME1 can account for the detected changes in the steady-state levels of 7S DNA when MGME1 expression is perturbed. However, this may be explained whether the length of 7S DNA is related to its stability. Early pulse-chase experiments aimed at determining the kinetics of D-loop synthesis and turnover found that longer 7S DNA species in mouse cells have the longest half-lives, suggesting that these are the most stable (43). Longer 7S DNA molecules, such as those seen in MGME1 siRNA samples and patient fibroblasts, would therefore be more stable and would increase in abundance. This is in line with the observed two-fold longer *in vivo* half-life time of 7S DNA in patient fibroblasts (Fig. 1B). Shorter 7S DNA molecules, such as those seen in cells overexpressing MGME1, would be less stable, consistent with their decreased abundance (Fig. 2B).

### Formation and persistence of the 11-kb sub-genomic linear dsDNA

In *MGME1* patient fibroblasts, a persistent sub-genomic linear dsDNA species was detected, the ends of which mapped to the two classically defined replication origins, O_H_ and O_L_ (Fig. 3). This species could also be generated from control human cultured cells by treatment with S1 nuclease, which degrades single-stranded DNA as well as nicked double-stranded DNA (Fig. 3). This linear species therefore appears to result from chromosome breakage of a theta-like replication intermediate, with ends at the replication origins. The fact that this linear dsDNA species can be generated in control cells indicates that site-specific stalling at the replication origins is a common occurrence in normal human cells, as reported previously for mouse cells (29). To our knowledge, this is the first time that such a DNA species has been reported in a case of human mitochondrial pathology.

The 11-kb sub-genomic dsDNA species in *MGME1* patient fibroblasts persists at levels of ∼20% of mtDNA under steady-state conditions. This is somewhat unexpected, because experiments in which a restriction endonuclease was targeted to mitochondria showed that linear mtDNA is rapidly eliminated (44), suggesting that mitochondria contain efficient mechanisms to remove linear dsDNA molecules. The presence of the 11-kb sub-genomic species under normal conditions would therefore imply that it is constantly produced in *MGME1* patient fibroblasts, likely as a by-product of replication. Consistent with this, our results show that high levels of sub-genomic major arc fragments accumulate in MGME1-null fibroblasts during the rapid phase of mtDNA proliferation that accompanies recovery from mtDNA depletion (Fig. 4D). An alternative explanation for the persistence of the sub-genomic fragment could be that MGME1 itself participates in the removal of linear mtDNA molecules, although given the strong preference of MGME1 towards ssDNA *in vitro* this appears less probable.

The generation and presence of this sub-genomic fragment could be a consequence of replication stalling *per se* as proposed previously (29), or alternatively, it is possible that programmed replication pausing occurs at O_L_ to provide a replication checkpoint. Such a checkpoint mechanism could, for example, (i) prevent excessively damaged partially replicated molecules from undergoing a full round of replication or (ii) assist HSP-initiated transcription to pass the replication fork, preventing possible head-to-head collisions between mtDNA transcription and replication. The first possibility would be consistent with MGME1 being a part of DNA repair mechanism(s), where the lack of MGME1 results in inability of the replisome to restart DNA synthesis after programmed replication stalling. This would be in line with data from the mutator mouse, where accumulation of a high number of point mutations in the nascent strand by the proofreading-deficient Polγ also leads to the generation of an analogous linear dsDNA species (28,29). According to the second option, MGME1 could play a role in preventing hazardous replication fork collisions with transcriptional bubbles, similarly to other ssDNA nucleases involved in replication of bacterial and nuclear genomes (45).

### Formation of unusual mtDNA rearrangements

Most deletions of mtDNA described so far have been observed in the major arc between the two classical replication origins of mtDNA (46–48). This has led to the assumption that slipped-strand replication is one of the main sources of mtDNA deletion generation (49). Replication can, however, also play a role in deletion formation through inducing double-strand breaks, generated at sites of stalled replication (50). If these linear DNA molecules are not eliminated, then repair processes, acting preferentially by homologous recombination, may convert them to biochemically intact but incomplete mtDNA molecules (27).

In contrast to the predominantly major arc deletions reported in the literature, tissue samples of *MGME1* patients exhibited (in addition to elevated levels of linear sub-genomic mtDNA molecules) a non-standard distribution of breakpoints of rearranged circular mtDNAs, such that: (i) classical major arc breakpoints are almost missing and (ii) 5′ starting positions were clustered in the minor arc around the tRNA^Phe^ gene, in the 12S ribosomal RNA gene and close to the tRNA^LeuUUR^ gene (Fig. 5A, Supplementary Material, Table S2). The vast majority of breakpoints therefore apparently seemed to remove more than two-thirds of the mitochondrial genome. More detailed analysis revealed, however, that the observed breakpoints originated not from classical deletions but from partial mtDNA duplications (Fig. 5B). Similar rearranged molecules have previously been observed in combination with classical deletions in heart muscle of healthy individuals (51). The fact that the breakpoints from the apparently O_L_-lacking molecules originate from replication competent duplications explains the observed clonal expansion of these rearranged mtDNA molecules. However, very surprisingly, breakpoints close to the O_L_ end of the prominent 11-kb linear sub-genomic fragment were completely lacking (Fig. 5A). Thus, the 11-kb linear sub-genomic fragment is either not recombinogenic *per se*, or alternatively, the mechanism allowing homologous recombination to occur is not properly functioning. As the first possibility does not explain the apparent lack of other major arc deletions, the latter possibility seems to be more likely. In this context, it is interesting to mention that the requirement of MGME1 for homologous recombination might be a consequence of its efficient 5′ end processing activity of ssDNA substrates, and a residual 5′ end processing activity of dsDNA substrates [cf. Fig. 3 in (25)]. It is possible that functional interactions with a helicase *in vivo* allow for unwinding dsDNA to expose longer stretch of ssDNA for resection. The finding of similarly rearranged mtDNA species in the skeletal muscle of a transgenic mouse model expressing a defective Twinkle^dup^ protein (52) might point towards Twinkle. As the generation of 3′ overhangs is a prerequisite for strand invasion during double-strand break repair by intramolecular homologous recombination (53,54), it is tempting to speculate that this missing activity might impair the formation of regular major arc breakpoints in *MGME1* patients. The irregular breakpoint hotspots observed in MGME1 deficiency could be accounted for by alternative forms of single-strand generation. Two hotspots of the detected duplication breakpoints are localized close to the D-loop, the triple-strand structure of which may facilitate single-strand annealing. The other hotspot was detected close to the tRNA^LeuUUR^ gene, which acts as a binding site for the mitochondrial transcription termination factor mTERF1 (31). The interaction of mTERF1 with mtDNA might also create the single-stranded DNA required for the strand invasion process during homologous recombination.

### Functional relationship between MGME1 and PolgA

Cells with compromised MGME1 activity (either patient fibroblasts or siRNA-transfected cells) share a number of molecular similarities with cells of the ‘mtDNA mutator’ mouse. This model animal carries a homozygous knock-in mutation in the *POLG* gene, encoding the catalytic subunit of the mtDNA polymerase, which inactivates the 3′ → 5′ exonucleolytic proofreading activity of the enzyme. On a molecular level, mutator mice accumulate extremely high levels of mtDNA point mutations and low levels of multiple mtDNA deletions (28,55,56). The similarities between MGME1-deficient and mutator mouse-derived cells include: (i) replication stalling at multiple sites throughout the mitochondrial genome, with a particular accumulation of forks in the NCR (25,29), (ii) chromosome breakage at O_H_ and O_L_, leading to the accumulation of an 11-kb linear dsDNA species [Fig. 3 and Fig. 4, (29)] and (iii) small tandem insertions or deletions, especially in the NCR region [Supplementary Material, Table S4, (34)]. These shared similarities, combined with the findings pointing towards a potential physical interaction of MGME1 with PolgA as shown by our proteomic approach followed by reciprocal pull-downs (Fig. 6), suggest that MGME1 and the 3′ → 5′ exonucleolytic activity of PolgA might be involved in the same process. Below we present some hypotheses regarding functional cooperation of MGME1 and PolgA.

In yeast, the 3′ → 5′ exonucleolytic activity of the lagging-strand nuclear DNA polymerase Polδ has been implicated in the removal of ssDNA flaps in Okazaki fragments (57,58). It is possible that the 3′ → 5′ exonucleolytic activity of PolgA is also necessary for DNA flap processing. If MGME1 is involved in the processing of 5′ ssDNA flaps arising from DNA synthesis, as was suggested previously (25), then many of these shared similarities could be accounted for. An alternative hypothesis relates to the capacity of the exonuclease-deficient Polγ to perform strand-displacement synthesis upon reaching an upstream DNA fragment. A general feature of exonuclease-deficient DNA polymerases is an increased strand-displacement activity, as has been observed for T7 DNA polymerase (59), T4 DNA polymerase (60), *Escherichia coli* DNA polymerase II (61) and yeast Polδ (62). Based on previous data, increased strand-displacement activity could also be a feature of the exonuclease-deficient Polγ. With this assumption, the shared similarities could result from the formation of longer DNA flaps by increased strand-displacement by the exonuclease-deficient Polγ, or by defects in the removal of DNA flaps in the case of MGME1 deficiency.

Little is known about what happens when forks meet to complete DNA replication in any organism studied thus far. The tandem insertions seen in the NCR of MGME1-deficient patient fibroblasts are reminiscent of control region multimers (CRMs) observed in the mutator mouse (34). It was suggested that CRMs might be indicative of problems with replication termination, which according to current models takes place in the NCR and may involve the exonuclease activity of Polγ (34). It is plausible that the loss of exonuclease activity of Polγ in the mutator mouse or in the MGME1-deficient cells perturbs mtDNA replication termination, manifested by multiplication of the NCR in both systems. This possibility would also additionally point towards a potential role of MGME1 in mtDNA replication termination.

In summary, we provide evidence that MGME1 activity is directly involved in the processing of 5′ ends of the 7S DNA. Furthermore, we show that loss of MGME1 leads to the accumulation of an 11-kb linear sub-genomic mtDNA fragment, a by-product of mtDNA replication originating from chromosome breakage at fragile sites. This is in agreement with its direct functional involvement in mtDNA replication, which is supported by the observed direct interaction of MGME1 with the mitochondrial replicase PolgA, suggesting that MGME1 is a constituent of the mitochondrial replisome. Therefore, MGME1 is a probable candidate to provide the replisome with a 5′ → 3′ exonuclease activity that is an inherent part of prokaryotic polymerases, but missing from their eukaryotic counterparts. In the poxvirus, FEN1-like nuclease, a 5′ → 3′ exonuclease that is necessary for seamless replication is also reported to be involved in repair of double-strand breaks (63). Similarly, the role of MGME1 in DNA maintenance is not necessarily restricted to replication but might also involve homologous recombination to remove undesirable free DNA ends. This latter role is supported by the abundance of irregular breakpoints in rearranged mtDNA molecules that are observed upon loss of MGME1 activity.

## MATERIALS AND METHODS

### Cell culture and transfection

HeLa cells were maintained in Dulbecco's Modified Eagle Medium (DMEM, Invitrogen) supplemented with 10% foetal calf serum (FCS, Thermo Scientific), 10 U/ml penicillin and 10 mg/ml streptomycin (Gibco). Fibroblast lines were cultured as mentioned above with the addition of 50 mg/ml uridine (Sigma). HEK293T Flp-In cells, following transfection, were maintained in DMEM supplemented with 10% tetracycline-free FCS (Biochrom AG), 10 U/ml penicillin and 10 mg/ml streptomycin (Gibco), 50 mg/ml hygromycin (Invitrogen) and 15 mg/ml blasticidin (Invivogen).

The construction of pcDNA5/FRT/TO encoding full-length cDNA MGME1 wild-type or K253A or PolgA with C-terminal FLAG and Strep2 tags was as described previously (25). HEK293T cells were stably transfected with the above-mentioned constructs as described previously (25). For overexpression analysis and the pull-downs as per Figure 6B, clonal HEK293T cell lines were induced to express MGME1 or its K253A variant with 20 ng/ml doxycycline for the indicated length of time. A stable cell line inducibly expressing MGME1.FLAG for the MS experiments was described previously (26).

For siRNA-mediated down-regulation experiments, 100 000 HeLa cells were transfected using Lipofectamine RNAiMAX (Invitrogen) with siRNA against MGME1 or negative control (Invitrogen Stealth) for 3 days and then re-transfected for a further 3 days. siRNA sequences used were as follows:

MGME1 siRNA 1: 5′ CGAGTCCTTCAGCAGACCATGACAA 3′
MGME1 siRNA 2: 5′ CCACGAAGCCTTGGAAAGCATACTT 3′

### Restriction mapping and Southern blotting

Total DNA was isolated from fibroblast cells using a DNeasy blood and tissue kit (Qiagen). Three micrograms of DNA was restricted using the indicated enzymes according to manufacturer's instructions (New England Biolabs). Where indicated, samples were subsequently treated with 60 U S1 nuclease (Promega) for 15 min at 37°C. Products were separated on 0.6% agarose gels (Invitrogen Ultrapure) and dry-blotted overnight onto nylon membrane (GE Magnaprobe). For high-resolution Southern blots, DNA was separated on 5% polyacrylamide gels and electroblotted in 0.5× TBE onto nylon membrane. Membranes were hybridized with appropriate radiolabelled probes overnight at 65°C in 0.25 m phosphate buffer (pH 7.6) and 7% SDS, then washed for 3 × 20 min in 1× SSC and 0.1% SDS and imaged using a phosphorimager (GE Healthcare) and scanned using a Typhoon 9410 scanner.

Primer sequences used for producing probes were as follows:

H1 probe fwd: 5′ TTACAGTCAAATCCCTTCTCGT 3′, nt16341–16362
H1 probe rev: 5′ GGATGAGGCAGGAATCAAAGACAG 3′, nt 128–151
mt2 probe fwd: 5′ CACTGAAAATGTTTAGACGGG 3′, nt 607–627
mt2 probe rev: 5′ GGCTCCTCTAGAGGGATATG 3′, nt 1185–1204
mt17 probe fwd: 5′ CCGTATTACTCGCATCAGG 3′, nt 9610–9628
mt17 probe rev: 5′ TTATGGAGAAAGGGACGC 3′, nt 10201–10218
18S probe fwd: 5′ GTTGGTGGAGCGATTTGTCT 3′
18S probe rev: 5′ GGCCTCACTAAACCATCCAA 3′

### Primer extension

Primers were 5′ radiolabelled using T4 Polynucleotide kinase (New England Biolabs) according to manufacturer's instructions, annealed to 1 μg total DNA and extended using Taq polymerase using 20 cycles of denaturation at 95°C and extension at 72°C. Sequencing ladders were produced using Sequenase polymerase according to manufacturer's instructions. Products were separated on 7 m urea, 5% polyacrylamide sequencing gels, dried and imaged using a phosphorimager (GE Healthcare).

### Antibodies

Primary antibodies used for western blotting were as follows: MGME1 (Sigma HPA040913, 1 : 500 dilution), β-actin (Sigma A2228, 1 : 300 000), PolgA (Santa Cruz, 1 : 1000), mtSSB1 (kindly donated by Dr Kang) and TFAM (kindly donated by Dr Wiesner). Secondary antibodies used were as follows: goat anti-rabbit HRP (Promega W401B, 1 : 2000) and goat anti-mouse HRP (Promega W402B, 1 : 2000).

### Induced mtDNA depletion and repopulation in human fibroblasts

Fibroblasts from 5 × 5 mm skin biopsies of *MGME1* patients and healthy controls were cultivated in DMEM medium supplemented with 10% foetal calf serum, 100 U/ml penicillin, 100 U/ml streptomycin, 0.05 mg/ml uridine and 2 mm glutamine in a 5% CO_2_ atmosphere at 37°C. Replication of mtDNA was inhibited in fibroblasts by treating cells with 20 µm ddC for 12 days, after which fibroblasts were grown in normal medium for another 20 days to enable mtDNA to repopulate the cells.

### PCR

Copy numbers of mtDNA were determined by quantitative PCR essentially as described previously (64). Briefly, a segment of the mtDNA in the minor arc was amplified using primers 3922F24 and 4036R26 (first number, 5′ nucleotide of primer; second number, length of primer; F, forward primer; R, reverse primer; numbering according to reference sequence NC_012920). A major arc segment was amplified with primers 14588F24 and 14695R20. Primers 16520F24 and 35R24 were used to amplify a short segment inside the 7S region of the mtDNA. *C*_T_ values were defined at the inflection points of fitted sigmoid curves (4-parameter Chapman curves) and were compared with those of the single copy nuclear gene *Kir4.1* amplified by primers KIR835F19 and KIR903R19 (numbering according to sequence U52155). Reactions were performed on a MyiQ qPCR system (Bio-Rad, Munich, Germany) using iQ SYBR Green Supermix (Bio-Rad) under the following conditions: 95°C for 7 min, and 45 cycles of 95°C for 15 s and 60°C for 1 min. 7S DNA copy numbers were calculated by subtracting the apparent copy number of the minor arc from apparent copy number of the 7S DNA region. Sub-genomic fragment copy numbers were calculated by subtracting minor arc values from the apparent copy numbers of the major arc. Minor arc copy number was considered as copy number of the complete mtDNA. All copy numbers were calculated for the total growing cell mass by multiplying per nucleus copy numbers by total cell numbers.

All other PCR techniques (single-molecule PCR, ligation-mediated PCR and long-extension PCR) are described in Supplementary Experimental Procedures.

### Pull-down experiments and mass spectrometry

The expression of the FLAG-tagged version of MGME1 was induced for 24 h with 25 ng/ml of tetracycline. Mitochondria isolation and preparation of mitochondrial protein extracts were performed as described previously (65) and are described in details in Supplementary Experimental Procedures. MS analysis was performed by LC–MS in the Laboratory of Mass Spectrometry (IBB PAS, Warsaw) using a nanoAcquity UPLC system (Waters) coupled to an LTQ-Orbitrap Velos mass spectrometer (ThermoScientific) as previously described (37), and the data were analysed as detailed in Supplementary Experimental Procedures. The pull-down experiments as per Figure 6B and C were performed as previously described (66).

## ETHICS STATEMENT

The study was approved by the Ethical Committees of the Universities of Bonn (186/09) and Milan. Written informed consent was obtained from all subjects.

## SUPPLEMENTARY MATERIAL

Supplementary Material is available at *HMG* online.

## FUNDING

This work was supported by the Medical Research Council, UK. This work was also supported by the Deutsche Forschungsgemeinschaft (KU 911/21-1 to W.S.K. and ZS 99/3-1 to G.Z.), the European Community (FP7 project EpiPGX, grant 279062 to W.S.K.) and the Grant from the Ministry of Science and Higher Education of Poland (to R.J.S., 0542/IP1/2011/71 Iuventus programme). Funding to pay the Open Access publication charges for this article was provided by the Medical Research Council, UK.

## Supplementary Material

Supplementary Data
