# Supplementary material for: Linear mtDNA fragments and unusual mtDNA rearrangements associated with pathological deficiency of MGME1 exonuclease
Source: Hum Mol Genet. 2014 Jun 30;23(23):6147–62. doi: 10.1093/hmg/ddu336 (PMC4222359; doi:10.1093/hmg/ddu336)
Supplement: Supplementary Data [file supp_23_23_6147__index.html]

Linear mtDNA fragments and unusual mtDNA rearrangements associated with pathological deficiency of MGME1 exonuclease — Linear mtDNA fragments and unusual mtDNA rearrangements associated with pathological deficiency of MGME1 exonuclease — Supplementary Data 

# Linear mtDNA fragments and unusual mtDNA rearrangements associated with pathological deficiency of MGME1 exonuclease

## Supplementary Data

Supplementary Data

**Files in this Data Supplement:**

- Supplementary Data - Pdf file
- Supplementary Table 5 - xlsx file
