# Supplementary material for: Linear mtDNA fragments and unusual mtDNA rearrangements associated with pathological deficiency of MGME1 exonuclease
Source: Hum Mol Genet. 2014 Jun 30;23(23):6147–62. doi: 10.1093/hmg/ddu336 (PMC4222359; doi:10.1093/hmg/ddu336)
Supplement: Supplementary Data [file supp_ddu336_ddu336supp.pdf]

Linear mtDNA fragments and unusual mtDNA rearrangements associated with pathological deficiency of MGME1 exonuclease

SUPPLEMENTARY INFORMATION

SUPPLEMENTARY FIGURES

Supplementary Figure 1 – 7S DNA levels in control and MGME1-null fibroblasts quantified by Southern blotting upon EtBr treatment

Supplementary Figure 2 – Ligation-mediated PCR mapping of free DNA ends in MGME1-deficient fibroblasts

Supplementary Figure 3 – Primer extension mapping of RNA-DNA 5’ ends in human control and MGME1-deficient patient fibroblasts.

Supplementary Figure 4 – Overexpression of MGME1 alters steady-state levels and length of 7S DNA

Supplementary Figure 5 – *In vitro* activity of MGME1 on bubble and D-loop DNA substrates

Supplementary Figure 6 – The 11-kb sub-genomic fragment lacks the minor arc of the mtDNA

Supplementary Figure 7 – mtDNA rearrangements in a patient with pathogenic POLG mutations

SUPPLEMENTARY TABLES

Supplementary Table 1 – Half-lives of mtDNA and sub-genomic fragments during ddC treatment of MGME1-deficient and control fibroblasts

Supplementary Table 2 – mtDNA breakpoints in patients with pathogenic MGME1 mutations

Supplementary Table 3 – mtDNA breakpoints in a patient with pathogenic POLG mutations

Supplementary Table 4 – Small tandem duplications in the non-coding region of mtDNA in skeletal muscle of patients with pathogenic MGME1 mutations

Supplementary Table 5 – Proteins co-purified with MGME1 identified by high resolution mass-spectrometry and quantified using label-free approach

SUPPLEMENTARY EXPERIMENTAL PROCEDURES

SUPPLEMENTARY REFERENCES

Supplementary Figure 1

7S DNA levels in control and MGME1-null fibroblasts quantified by Southern blotting upon EtBr treatment

(A) Southern blot analysis of control and MGME1-null fibroblasts (FB1976) upon treatment with EtBr (concentration) for the indicated time. Total DNA preparations were restricted using *Eco*RI and *Bam*HI and hybridised with a probe specific for the NCR of human mtDNA (mtDNA region: 16,341–151; detecting both restriction fragment-length mtDNA and 7S DNA). **(B)** Quantification of 7S DNA signal from Southern blots as per (A). Error bar =1SD.

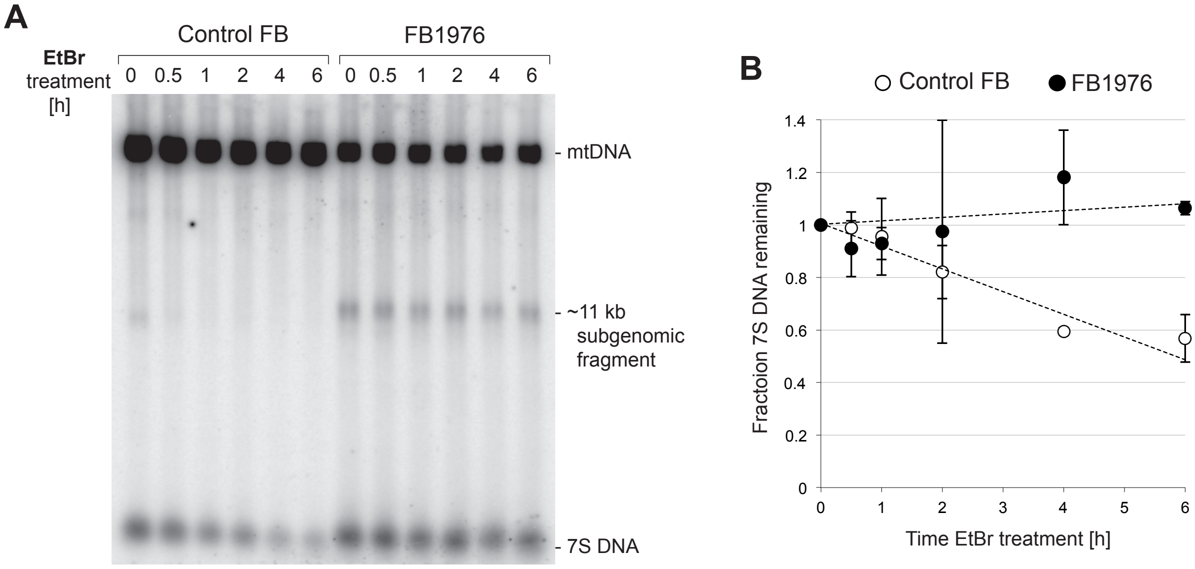

Supplementary Figure 2

Ligation-mediated PCR mapping of free DNA ends in MGME1-deficient fibroblasts

Representative sequencing chromatograms of single molecule PCR products from ligation-mediated PCR (LM-PCR) are shown. Grey areas indicate the end of the linker sequence. **(A)** Mapping of the 5' ends of 7S DNA after T4 polymerase pre-treatment. For amplification, an mtDNA-specific primer located within the 7S DNA region was used. **(B)** Determination of the 3' ends of 7S DNA after mung bean nuclease pre-treatment. **(C)** Ends of the 11-kb sub-genomic fragment in the vicinity of the H-strand replication origin (O_H, upper panel) and the L-strand replication origin (O_L, lower panel). The linker was ligated to blunt double-stranded ends without pre-treatment. The mtDNA-specific primer for the amplification of O_H ends located outside of the 7S DNA region.

Supplementary Figure 2 - continued

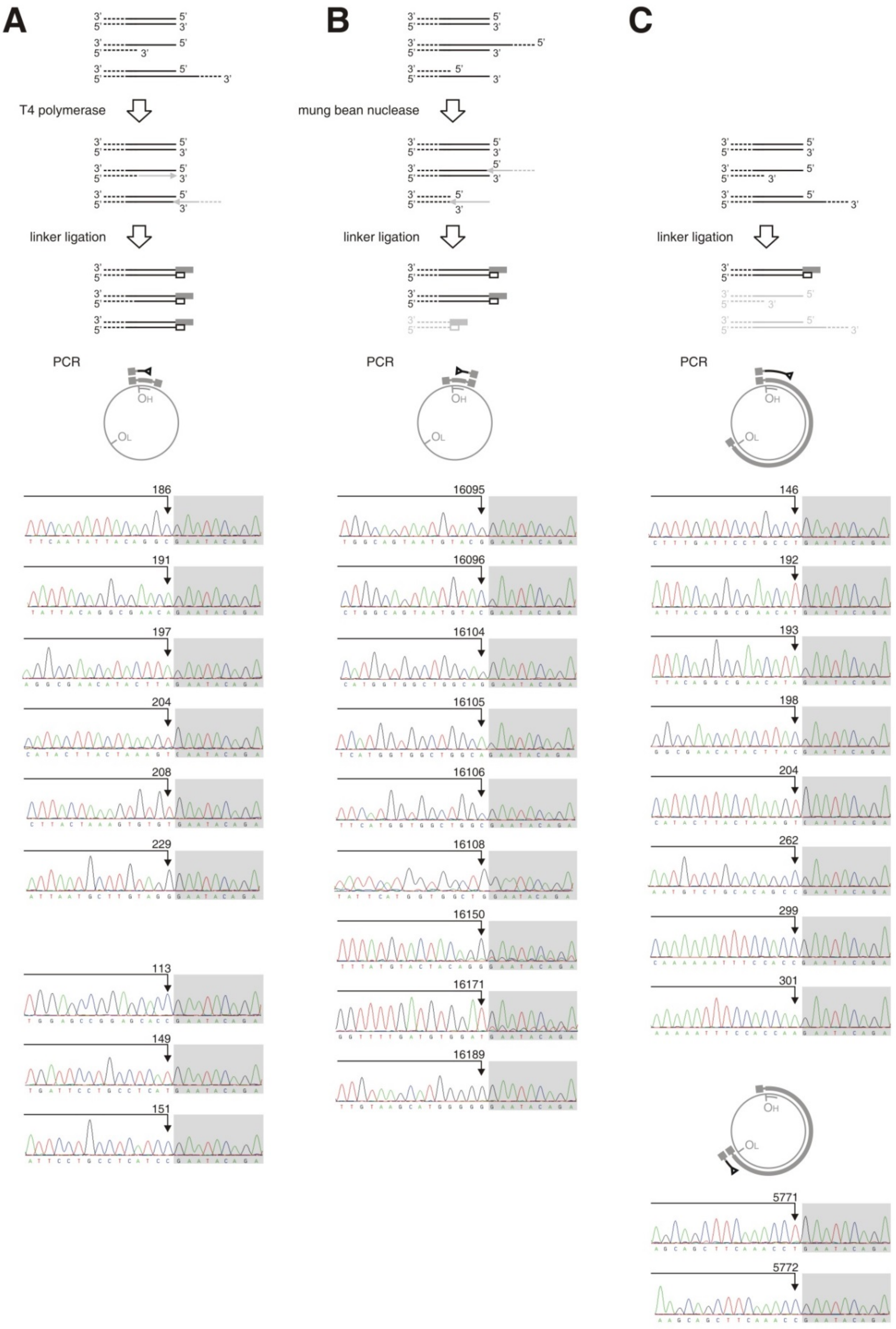

Supplementary Figure 3

Primer extension mapping of RNA-DNA 5’ ends in human control and MGME1-deficient patient fibroblasts

(A) Reverse transcription activity of Taq polymerase. PEx was performed on ssRNA-DNA chimeric substrates (RNA8-DNA25 and RNA15-DNA18) or ssDNA (DNA33) using a radioactively labelled, 17-nt-long primer as indicated (top). Reactions were analysed by 15 % urea PAGE and subjected to autoradiography. **(B)** PEx was performed using total DNA preparations extracted from human control and patient fibroblasts (FB1976), which were not initially treated with RNase A, using a radioactively labelled primer annealing at nt position 16,569–18 of the human mtDNA. In the last two lanes the same analysis was performed using samples pre-treated with RNaseH1. **(C)** siRNA downregulation of MGME1 in human cells. Western blot of total HeLa cell lysate transfected with two different siRNAs to *MGME1* for 3 and 6 days as indicated. siRNA to GFP was used as a transfection control. β-actin serves as a loading control.

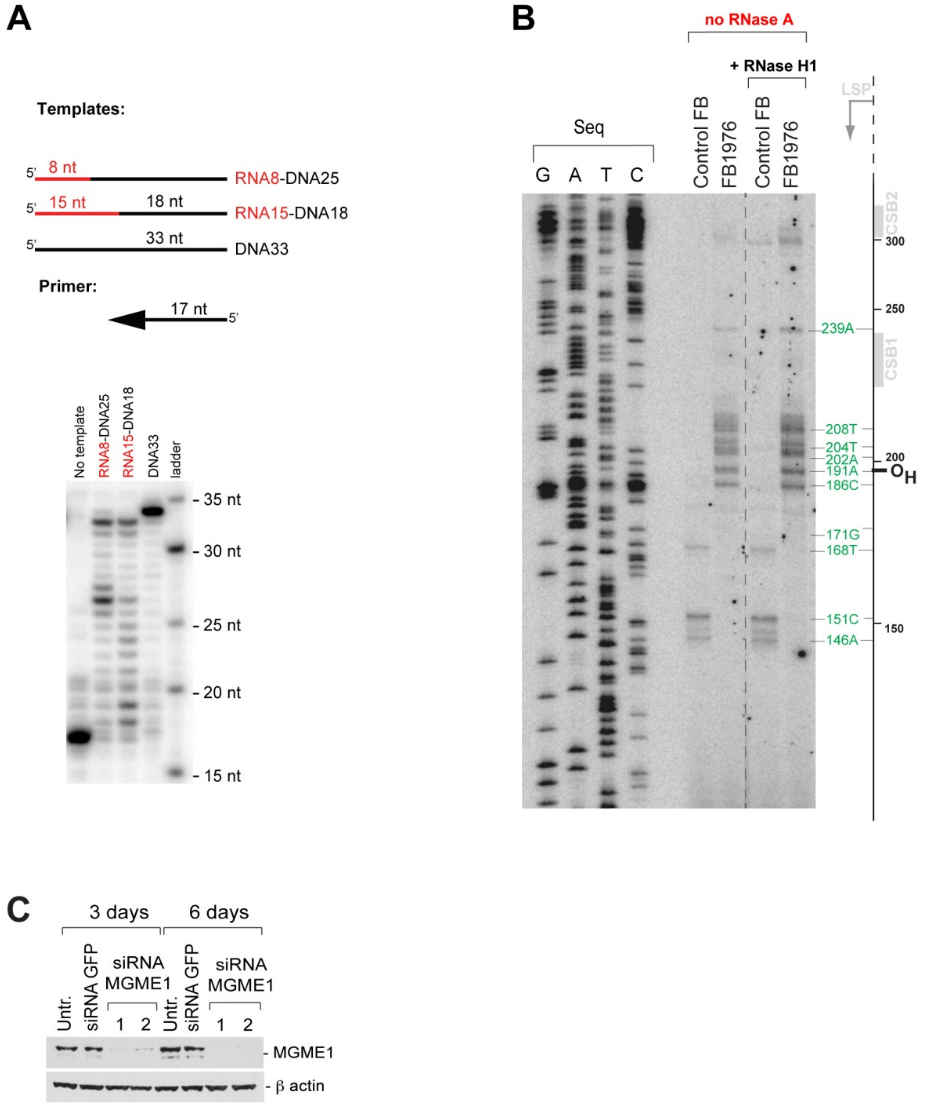

Supplementary Figure 4

Overexpression of MGME1 alters steady-state levels and length of 7S DNA

(A) Western blot (short and long exposures) of HEK293T cells expressing WT MGME1 under a doxycycline inducible promoter (uninduced and induced cells), control fibroblasts, and fibroblasts transduced with WT MGME1 using two different lentiviral titers (1x and 10x) as previously described (1). Note: Flag.Strep2 tag causes an increase in molecular weight of MGME1 in HEK293T cells **(B)** Total DNA from cells expressing the wild-type version of MGME1 (left) or the K253A catalytic mutant (right) in a 10-day time course was restricted using *Eco*RI and *Bam*HI and analysed by 1D Southern blotting using a probe specific for the NCR of human mtDNA (mtDNA region: 16,341–151; detecting both restriction fragment-length mtDNA and 7S DNA). **(C)** Mapping of DNA 5' ends in the human mtDNA NCR of HEK293T cells expressing the wild-type version of MGME1 (left) or the K253A catalytic mutant (right) in a 10-day time course using primer extension (PEx). A radioactively-labelled primer annealing at nt position 16,569–18 of the human mtDNA was extended using a thermal cycler. Samples were analysed on a 4 % denaturing polyacrylamide gel and subjected to autoradiography. A sequencing reaction from the same primer was run alongside in order to identify the PEx bands. H, non-transfected control. CSB, conserved sequence block. LSP, light-strand promoter.

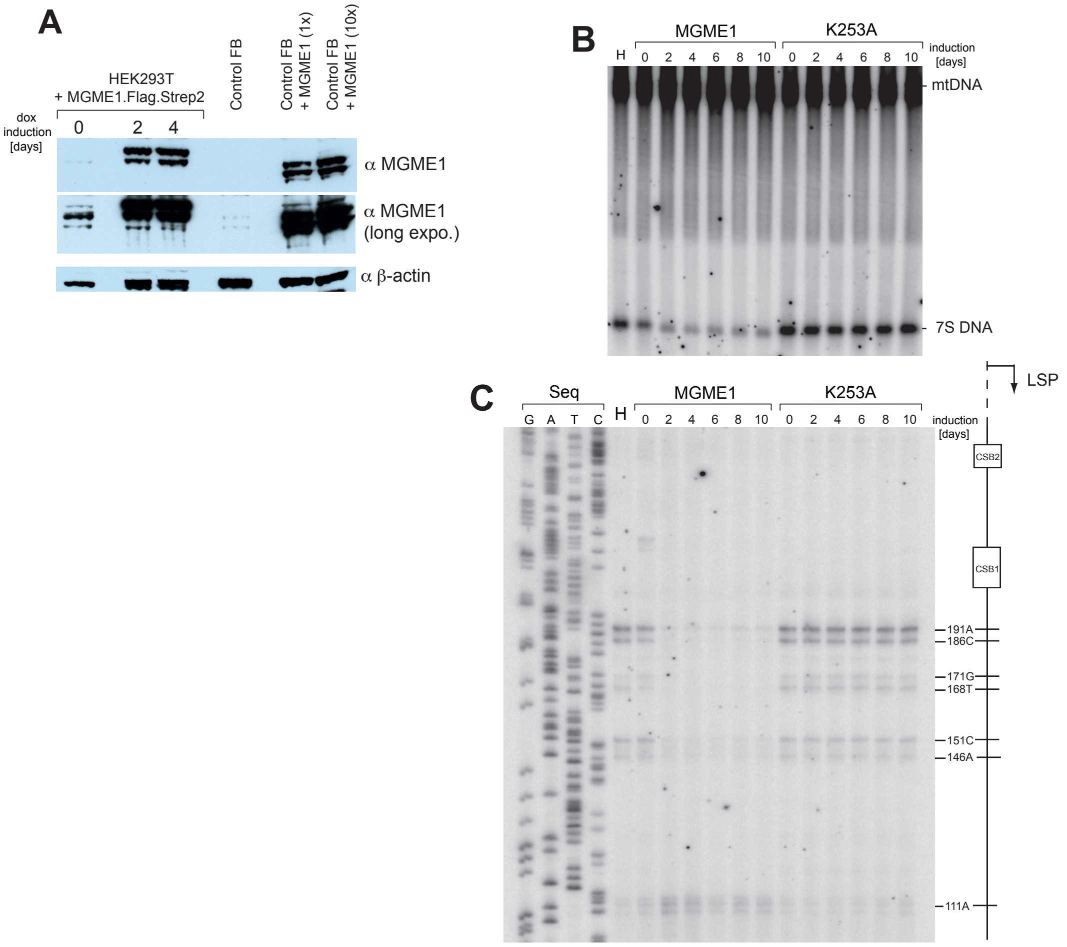

Supplementary Figure 5

***In vitro* activity of MGME1 on bubble and D-loop DNA substrates**

(A) *In vitro* activity of recombinant MGME1 on ssDNA, DNA bubble substrates and D-loop-containing substrates. 1 pmol of radioactively labeled substrates (asterisks in the schemes indicate the label) were incubated for 30 min with increasing concentrations (0.125, 0.25, 0.5, 1, 2, 4 pmol) of purified MGME1 or 4 pmol of the K253A mutant. Reaction products were subjected to denaturing PAGE and autoradiography. **(B)** Quantification of the reactions with PAGE and autoradiography as per (A). Substrate band intensity was quantified using ImageQuant software and plotted against enzyme concentration. **(C)** The strategy of constructing the substrates and sequences of the oligonucleotides used.

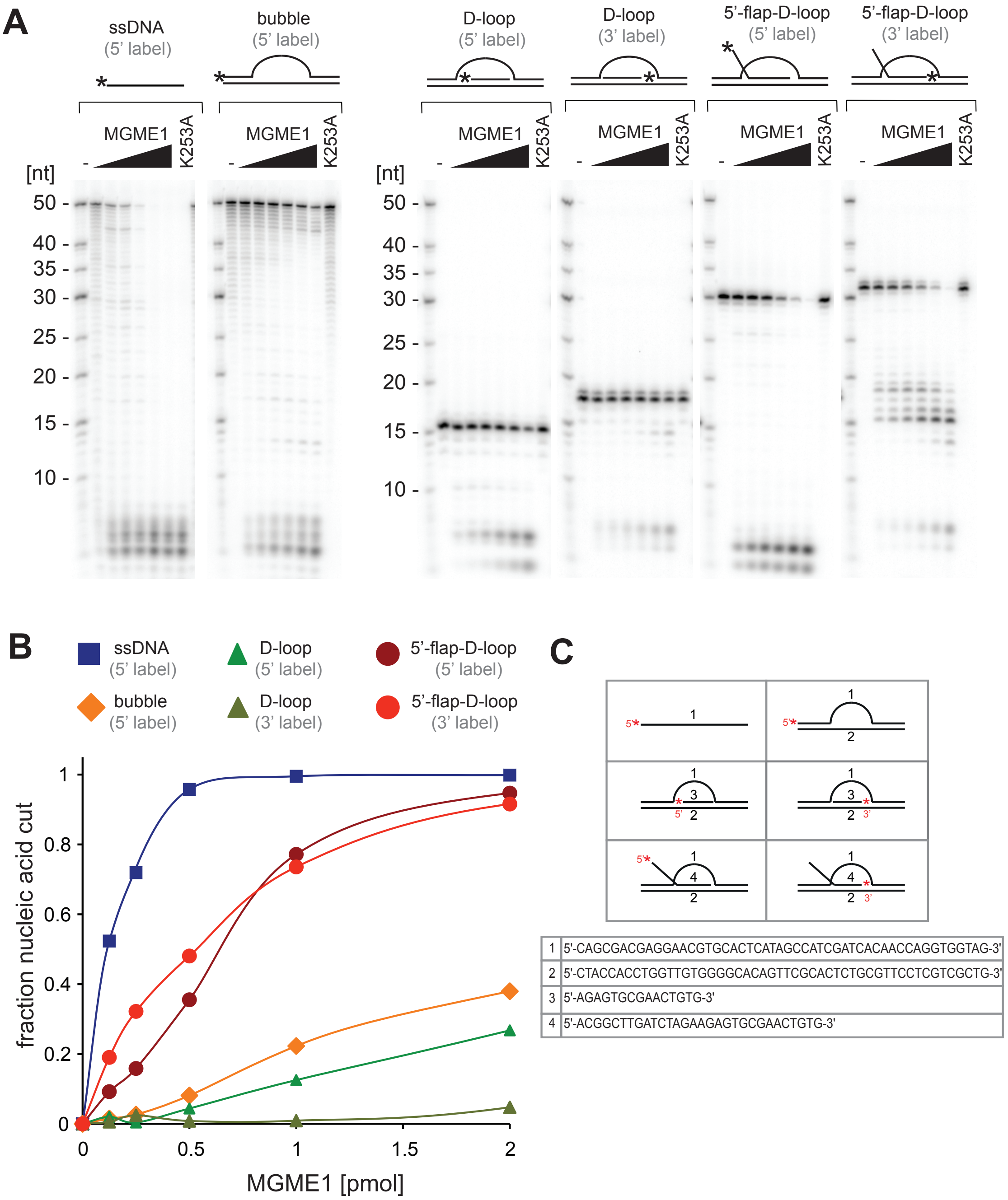

1	5'-CAGCGACGAGGAACGTGCACTCATAGCCATCGATCACAACCAGGTGGTAG-3'
2	5'-CTACCACCTGGTTGTGGGGCACAGTTCGCACTCTGCGTTCCTCGTCGCTG-3'
3	5'-AGAGTGCGAACTGTG-3'
4	5'-ACGGCTTGATCTAGAAGAGTGCGAACTGTG-3'

Supplementary Figure 6

The 11-kb sub-genomic fragment lacks the minor arc of the mtDNA

(A) Restriction mapping for the 11-kb sub-genomic fragment of human mtDNA as per **Figure 3B** of the main text, but using a DNA probe located in the minor arc of the mtDNA (nt 607–1,204). The black bar indicates the probe binding site. Note the absence of shorter bands, as opposed to Figure 3B.
(B) Digested FB1976 mtDNA retains a proportion of 7S DNA unless heat treated. 6 µg of total FB1976 DNA was restricted using AflII/XhoI or AflII/BamHI, then half of the digested product was heated at 95 °C for 5 minutes. Products were separated on a 0.6 % agarose gel, blotted and probed for the D-loop region. The restriction product present immediately above the expected 6.4 kb restriction fragment (indicated by an asterisk) is eliminated by heat treatment. This species is believed to represent restricted mtDNA that still contains a stably-bound 7S DNA molecule, based upon its gel mobility and elimination by heat treatment.

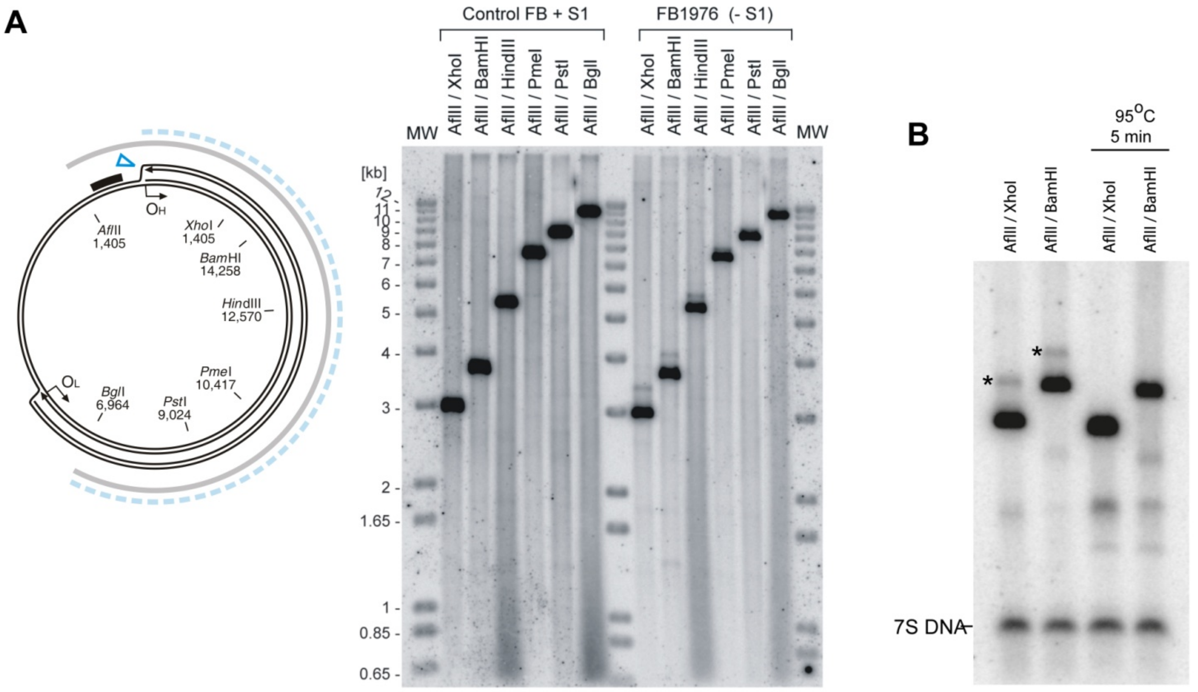

Supplementary Figure 7

mtDNA rearrangements in a patient with pathogenic POLG mutations

Circos representation of detected mtDNA breakpoints in a patient compound heterozygous for pathogenic POLG mutations W748S and L752P. Arrowheads indicate the orientation of the deletions. The part of the genome that is deleted spans counter clockwise from the root to the head of the arrow (as indicated by dashed line in the panel "del" of the scheme on the right). A detailed list of the detected breakpoints is available in **Suppl. Table 3**. Breakpoints that conform to regular major-arc deletions are marked in blue, grey arcs indicate breakpoints that remove O_L. wt, wild-type; del, deleted mtDNA molecules.

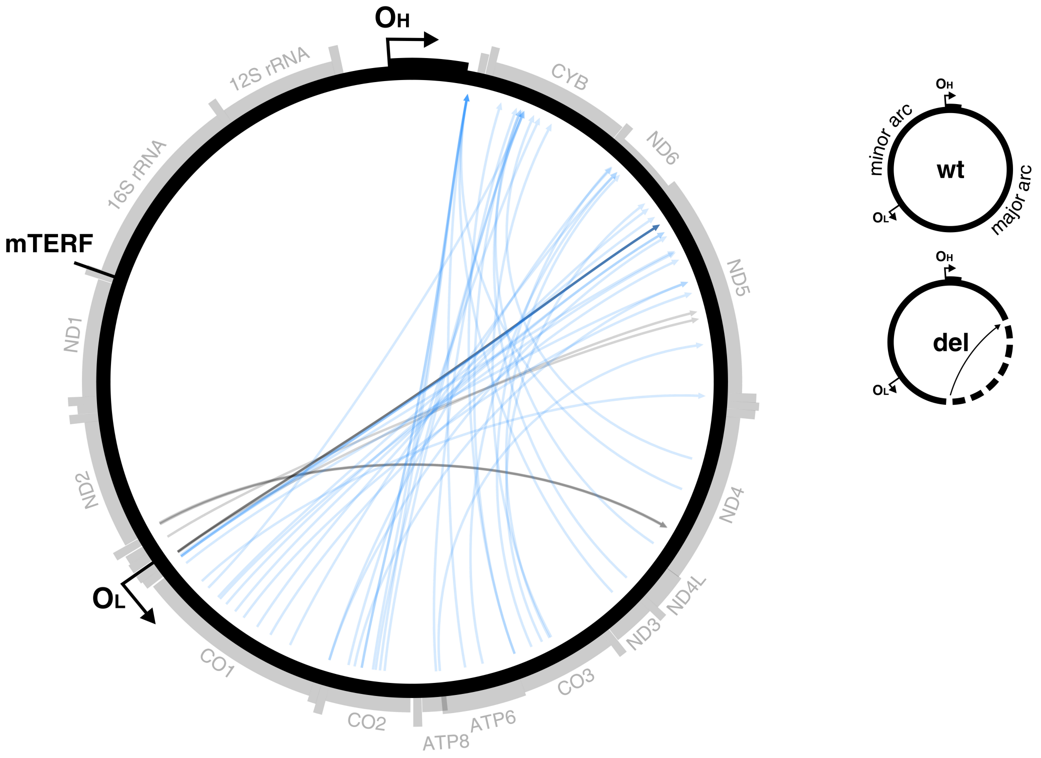

Supplementary Table 1

Half-lives of mtDNA and sub-genomic fragments during ddC treatment of MGME1-deficient and control fibroblasts

Half-life times (in days) were determined by nonlinear regression analysis of qPCR data assuming simple exponential decay kinetics. 7S DNA, the 11-kb sub-genomic fragment, and complete mtDNA amounts were calculated as described in Methods. Two controls and two patient samples were investigated, each determined in three independent reactions. [a,b], significant difference, $p < 0.01$ (t test).

Region	Controls	Patients
7S DNA	0.99 ± 0.47[a]	4.08 ± 1.26[a]
mtDNA	2.95 ± 1.72	3.36 ± 0.42[b]
11-kb DNA	–	6.78 ± 1.98[b]

Supplementary Table 2

mtDNA breakpoints in patients with pathogenic MGME1 mutations

Sample	Start	Stop	Repeat length[b]	Count	PrimerF	PrimerR
M2061	262	16069		2	10F	16496R
	461	13522			10F	16496R
	529	14382			10F	16496R
	3285	16069			3137F	45R
	3959	15737	7	4*	3137F	45R
	3615	12986	11	1*	3137F	45R
M3737	315	16193	10		10F	16496R
	499	15028			10F	16496R
	508	14808			10F	16496R
	512	13925	6		10F	16496R
	535	14119			10F	16496R
	873	15539		5	10F	16496R
	886	15972		4	10F	16496R
	3231	13472			3137F	45R
	3296	16034		4	3137F	45R
	3558	14816		2	10F	16496R
	3578	14813	8	1*	10F	16496R
	3743	15636		3	3137F	45R
	4514	14310		4	3137F	45R
	5438	13792			3137F	45R
	14287	16264	7		14066F	16503R
M1976	3258	16070		3	3137F	45R
	3846	14701			10F	16496R
	4490	11905			3137F	45R
	5240	13812			3137F	45R
FB1976	84	16094			10F	16496R
	168	16222			10F	16496R
	196	15195			10F	16496R
	399	16061			10F	16496R
UR1976	3248	15972			3137F	45R
	3272	15541			3137F	45R
	3321	15965		2	3137F	45R
	3346	15971			3137F	45R
	3460	14389			3137F	45R
	3615	12986	11	2*	3137F	45R
	4902	15848	7		3137F	45R
	8649[a]	16084[a]	12		3137F	45R
M931	115	12304			641F	15623R
	308	13786	5		641F	15623R
	310	15539			641F	15623R
	462	13788	6		641F	15623R
	482	14142	6		641F	15623R
	499	14517		2	641F	15623R
	506	14346	6		641F	15623R
	540	4352			16520F	4833R
	541	14396			641F	15623R

continued

Sample	Start	Stop	Repeat length	Count	PrimerF	PrimerR
M931	561	13785		3	641F	15623R
	562	13462		2	641F	15623R
	791	15515		2	641F	15623R
	887	16079			10F	16496R
	899	16115	11	2	10F	16496R
	3266	16078			3137F	45R
	3273	16071		5	3137F	45R
	3280	16032		3	3137F	45R
	3572	14812			3137F	45R
	3578	15546	10	1*	3137F	45R
	3586	15641			3137F	45R
	3603	14801			641F	15623R
	3611	16076		2	3137F	45R
	3614	14068			3137F	45R
	4228	14832			3137F	45R
M4050	539	14393	10		10F	16496R
	561	15529	6	5	10F	16496R
	1228	14532			10F	16496R
	3529	15749			3137F	45R
	4468	14824			3137F	45R
	5742	14586			3137F	45R
FB4050	971	15212			10F	16496R
M4052	453	15197		5	10F	16496R
	461	14429		4	10F	16496R
	482	13775			10F	16496R
	515	12562	7		10F	16496R
	899	16079			10F	16496R
	3333	14138	9		3137F	45R
	3475	13626			3137F	45R
	3496	13039			3137F	45R
	3602	15272	5		3137F	45R
	3615	12986	11	1*	3137F	45R
	3721	15945		2	3137F	45R
	3959	15737	7	1*	3137F	45R
	4773	16320			3137F	45R
FB4052	316	16193			10F	16496R

Breakpoints were determined by sequencing single-molecule PCR products that were amplified using the indicated primers. 'Start' and 'Stop' indicate the first and last deleted nucleotide positions, respectively. Count values are indicated if breakpoints were detected more than once. [a], the only breakpoint that conforms to regular major-arc deletions; [b], direct repeat lengths of at least 5 nucleotides are indicated; *, breakpoints detected in more than one individual; M, skeletal muscle; FB, fibroblasts; UR, urine sediment; PrimerF, forward primer used for single-molecule PCR amplification; PrimerR, reverse primer.

Supplementary Table 3

mtDNA breakpoints in a patient with pathogenic POLG mutations

Sample	Start	Stop	Repeat length[a]	Count	PrimerF	PrimerR
M1763	5477	11061	5	2	3137F	45R
	5484	11056	7	2	3137F	45R
	5491	11061		2	3137F	45R
	5623	13000	11		3137F	45R
	5788	13922			3137F	45R
	5788	13067		2	3137F	45R
	5789	13922			3137F	45R
	5790	13922			3137F	45R
	5796	13922			3137F	45R
	5830	13922			3137F	45R
	5841	13649		2	3137F	45R
	5841	13923			3137F	45R
	5842	13922			3137F	45R
	5918	13344			3137F	45R
	6135	12302			3137F	45R
	6219	15538	6		3137F	45R
	6342	14004	11		3137F	45R
	6366	13635	5	2	3137F	45R
	6440	13841			3137F	45R
	6544	13843	8		3137F	45R
	6640	13240			3137F	45R
	6789	14098			3137F	45R
	6930	13563	10		3137F	45R
	7132	14533	7	2	3137F	45R
	7515	14594			3137F	45R
	7516	13799			3137F	45R
	7695	15386			3137F	45R
	7752	13346		2	3137F	45R
	7818	16071		2	3137F	45R
	7819	16070	6		3137F	45R
	7922	16071			3137F	45R
	7946	15539			3137F	45R
	7986	16072			3137F	45R
	8029	15541	5		3137F	45R
	8498	15612	13		3137F	45R
	8533	12766			3137F	45R
	8766	16068		2	3137F	45R
	8929	14530	4		3137F	45R
	9233	15572			3137F	45R
	9233	15760			3137F	45R
	9399	14603			3137F	45R
	9505	14155			3137F	45R
	9582	14498	6		3137F	45R
	9598	15267	8		3137F	45R

continued

Sample	Start	Stop	Repeat length[a]	Count	PrimerF	PrimerR
M1763	10292	16070			3137F	45R
	10477	15556		2	3137F	45R
	10874	15572			3137F	45R
	11432	16072			3137F	45R
	11725	15443	5		3137F	45R

Breakpoints were determined by sequencing single-molecule PCR products that were amplified using the indicated primers. 'Start' and 'Stop' indicate the first and last deleted nucleotide positions, respectively. Count values are indicated if breakpoints were detected more than once. [a], direct repeat lengths of at least 5 nucleotides are indicated; PrimerF, forward primer used for single-molecule PCR amplification; PrimerR, reverse primer.

Supplementary Table 4

Small tandem duplications in the non-coding region of mtDNA in skeletal muscle of patients with pathogenic MGME1 mutations

Sample	NCR duplications*	PrimerF	PrimerR
M3737	–16122/16070–16125/16267–16553/29–	16099F	136R
	–16245/16083–16132/16348–	16099F	136R
M931	–3272/16072–16137/16069–16336/16311–	3137F	45R
	–3272/16072–16137/16069–16336/16311–	3137F	45R
	–3272/16072–16137/16069–	3137F	45R
	–3272/16072–16137/16069–	3137F	45R
	–3265/16079–16134/16084–	3137F	45R
	–3610/16077–16151/16074–	3137F	45R
	–898/16116–16149/16075–	10F	16496R
	–898/16080–16117/16082–	10F	16496R
	–898/16116–16128/16082–	10F	16496R

* Breakpoints of tandem duplications are indicated by slashes surrounded by the positions of the first and last retained nucleotides; dashes indicate continuous sequences. PrimerF, forward primer used for single-molecule PCR amplification; PrimerR, reverse primer.

SUPPLEMENTARY EXPERIMENTAL PROCEDURES

Single-molecule PCR

To identify specific breakpoints in the mitochondrial genome, a single-molecule PCR approach was used as described previously (2). Single mtDNA breakpoints were amplified in 42 cycles of PCR using TaKaRa LA Taq Hot Start polymerase and primers 3137F26 and 45R22, primers 10F and 16469R, or primers 15974F23 and 15623R20. Total template DNA was diluted to degrees, where only a part of multiple identical reactions resulted in amplification products (ideally less than 50 %). It is reasonable to assume that, under these conditions, each positive reaction originated from a single mtDNA molecule. Deletion breakpoints were mapped by re-amplifying single-deletion amplicons using diverse primer pairs located within the amplified region and direct sequencing of re-amplified products.

Ligation-mediated PCR

To determine free ends of linear mtDNA molecules, we used a modified version of the ligation-mediated PCR method (3). An asymmetric one-side blunt-end double-stranded DNA adaptor was created by annealing two non-phosphorylated oligonucleotides of different sizes, as described by Kang et al. [23]. 100 pmole of this linker was ligated with 0.2 μg of sample DNA using T4 ligase (New England Biolabs) in a volume of 60 μl. Amplification of the ligated strand was done using an mtDNA-specific and an adaptor-specific primer, the latter at 1/8 concentration of the former. To map the exact ends of mtDNA fragments, we performed the amplification under single-molecule conditions. Single amplicons were then sequenced, and end points were defined as the last mtDNA position before the first nucleotide of the

adaptor sequence (**Suppl. Figure 2**). Since not all linear molecules of interest were blunt-ended *in vivo*, we optionally pre-treated DNA samples to create ligatable blunt ends. To investigate 5' ends of the 7S DNA we pre-treated samples with T4 DNA polymerase (New England Biolabs) that filled in the 3' end of the opposite strand if it was shorter, and removed if it was a 3' overhang (**Suppl. Figure 2A**). 3' ends of the 7S DNA were investigated on DNA samples that were pre-treated with mung bean nuclease (New England Biolabs) that removes overhangs (**Suppl. Figure 2B**). Depending on the region to be investigated, mitochondrial primers varied. To investigate 5' 7S DNA ends primer 16413R19 was used, for 3' 7S DNA ends primer 10F31, for O_H ends of the 11-kb sub-genomic fragment primer 15974F23, and for the O_L ends primer 5985R25.

Long extension PCR

To investigate the presence of duplicated mtDNA molecules, we used a PCR-based method, similar to that reported by Williams et al (4). Primers 1056F22 and 1144R22 were used to amplify a short mtDNA fragment by the following steps: 2.5 min at 95°C; 10 cycles of 20 s at 92°C and 14 min at 68°C; 20 cycles of 25 s at 92°C and 16 min at 68°C; finally 10 min followed at 72°C. The extended elongation time enabled the polymerase to amplify fragments of several thousand base pairs in size. Such long molecules can be amplified in addition to the short fragments if more than one copy of the primer-binding region are present on some of the mtDNA molecules (**Figure 5B**, main text). PCR products were analysed on an ethidium-bromide stained agarose gel.

Pull-down experiments and mass spectrometry

Expression of the FLAG-tagged version of MGME1 was induced for 24 hours with 25 ng/ml of tetracycline. Mitochondria isolation and preparation of mitochondrial protein extracts were performed as described previously (5). After clearing by centrifugation, protein extracts were mixed with anti-FLAG M2 beads (Sigma) and rotated for 2 h at 4°C. After incubation, resins were washed extensively with ice-cold IPP 150 solution (10 mM Tris-HCl pH 8.0, 150 mM NaCl, 0.1 % TritonX-100) and then with ice-cold TEV cleavage buffer (10 mM Tris-HCl pH 8.0, 150 mM NaCl, 0.5 mM EDTA, 1 mM DTT). Proteins were eluted by treatment with TEV protease for 2 h at 18°C, precipitated with pyrogallol red (6) and analyzed by mass spectrometry. Protein pellets were dissolved in 50 µl of 100 mM NH_4HCO_3 and subjected to a standard procedure of trypsin digestion: proteins were reduced with DTT (10 mM) for 30 min at 57°C, alkylated with iodoacetamide (50 mM) in darkness for 45 min at room temperature, and digested overnight at 37°C with 10 ng/µl trypsin. MS analysis was performed by LC-MS in the Laboratory of Mass Spectrometry (IBB PAS, Warsaw) using a nanoAcquity UPLC system (Waters) coupled to an LTQ-Orbitrap Velos mass spectrometer (ThermoScientific) as previously described (7). MS raw data were analyzed and quantified using Andromeda/MaxQuant (8) software (version 1.3.0.5). Samples were searched against a randomized protein database (Uniprot, organism: human, protein status: reviewed, 22 732 entries, 18 May 2012). A "common contaminants" database (incorporated in MaxQuant software) containing commonly occurring contaminations (like keratins) was employed during search. Peptide m/z signal intensities were calculated by summing intensities over the entire elution chromatogram of the peptide, and protein intensity were calculated as the sum

of intensities for all peptides for the given protein. For the purpose of analysis, if a given protein was not detected in a sample then its intensity was arbitrarily set to 1. MaxQuant software was used to match peptides between MS runs to deal with the possible problem of insufficient sequencing during LC-MS runs and for accurate ranking of the identified proteins according to MS signal intensity. Matching between runs is based on comparing peptide identification from any of the LC-MS runs in which the peptide has been identified by MS/MS to another LC-MS run where no MS/MS spectrum has been acquired for that MS peptide feature or the peptide could not be identified based on the MS/MS spectrum.

Protein purification and in vitro activity assays

Clonal HEK293T cells were induced to overexpress MGME1.Flag.Strep2 using 50 ng/ml doxycycline for 48 hours. Mitochondria were isolated from cells using a hypotonic lysis and differential centrifugation procedure as described previously (9), and MGME1 protein isolated from mitochondria using a Strep-tactin gravity flow column (IBA) as described previously (1). 1 pmol of labelled DNA substrate was incubated with incremental concentrations of MGME1 protein for 30 min at 37 ^{o}C in a reaction buffer containing 10 mM tris pH 7.6, 20 mM $MgCl_2$, 1 mM DTT and 0.1 mg/ml BSA. Reactions were then frozen on dry ice and separated on 10 % polyacrylamide, 7 M urea gels, dried and imaged using a phosphorimager.

SUPPLEMENTARY REFERENCES

1. Kornblum, C., Nicholls, T.J., Haack, T.B., Scholer, S., Peeva, V., Danhauser, K., Hallmann, K., Zsurka, G., Rorbach, J., Iuso, A., Wieland, T., Sciacco, M., Ronchi, D., Comi, G.P., Moggio, M., Quinzii, C.M., DiMauro, S., Calvo, S.E., Mootha, V.K., Klopstock, T., Strom, T.M., Meitinger, T., Minczuk, M., Kunz, W.S., and Prokisch, H. (2013) Loss-of-function mutations in MGME1 impair mtDNA replication and cause multisystemic mitochondrial disease. *Nat Genet*, **45**, 214-9.
2. Zsurka, G., Baron, M., Stewart, J.D., Kornblum, C., Bos, M., Sassen, R., Taylor, R.W., Elger, C.E., Chinnery, P.F., and Kunz, W.S. (2008) Clonally expanded mitochondrial DNA mutations in epileptic individuals with mutated DNA polymerase gamma. *J Neuropathol Exp Neurol*, **67**, 857-66.
3. Kang, D., Miyako, K., Kai, Y., Irie, T., and Takeshige, K. (1997) In vivo determination of replication origins of human mitochondrial DNA by ligation-mediated polymerase chain reaction. *J Biol Chem*, **272**, 15275-9.
4. Williams, S.L., Huang, J., Edwards, Y.J., Ulloa, R.H., Dillon, L.M., Prolla, T.A., Vance, J.M., Moraes, C.T., and Zuchner, S. (2010) The mtDNA mutation spectrum of the progeroid Polg mutator mouse includes abundant control region multimers. *Cell Metab*, **12**, 675-82.
5. Szczesny, R.J., Borowski, L.S., Brzezniak, L.K., Dmochowska, A., Gewartowski, K., Bartnik, E., and Stepien, P.P. (2010) Human mitochondrial RNA turnover caught in flagranti: involvement of hSuv3p helicase in RNA surveillance. *Nucleic Acids Res*, **38**, 279-98.
6. Aguilar, R.M., Bustamante, J.J., Hernandez, P.G., Martinez, A.O., and Haro, L.S. (1999) Precipitation of dilute chromatographic samples (ng/ml) containing interfering substances for SDS-PAGE. *Anal Biochem*, **267**, 344-50.
7. Orlowska, K.P., Klosowska, K., Szczesny, R.J., Cysewski, D., Krawczyk, P.S., and Dziembowski, A. (2013) A new strategy for gene targeting and functional proteomics using the DT40 cell line. *Nucleic Acids Res*, **41**, e167.
8. Cox, J. and Mann, M. (2008) MaxQuant enables high peptide identification rates, individualized p.p.b.-range mass accuracies and proteome-wide protein quantification. *Nat Biotechnol*, **26**, 1367-72.
9. Rorbach, J., Nicholls, T.J., and Minczuk, M. (2011) PDE12 removes mitochondrial RNA poly(A) tails and controls translation in human mitochondria. *Nucleic Acids Res*, **39**, 7750-63.
